# LC-ESI-HRMS — lipidomics of phospholipids: Characterization of extraction, chromatography and detection parameters

**DOI:** 10.1007/s00216-023-05080-0

**Published:** 2024-01-12

**Authors:** Katharina M. Rund, Laura Carpanedo, Robin Lauterbach, Tim Wermund, Annette L. West, Luca M. Wende, Philip C. Calder, Nils Helge Schebb

**Affiliations:** 1 Chair of Food Chemistry, Faculty of Mathematics and Natural Sciences, University of Wuppertal, Gaussstr. 20, 42119 Wuppertal, Germany; 2 School of Human Development and Health, Faculty of Medicine, University of Southampton, Southampton, UK; 3 National Institute for Health Research (NIHR) Southampton Biomedical Research Centre, University Hospital Southampton NHS Foundation Trust and University of Southampton, Southampton, UK

**Keywords:** Phospholipids, Reversed-phase liquid chromatography, High-resolution mass spectrometry, Untargeted analysis, Ion suppression, n3-PUFA supplementation

## Abstract

Lipids are a diverse class of molecules involved in many biological functions including cell signaling or cell membrane assembly. Owing to this relevance, LC–MS/MS-based lipidomics emerged as a major field in modern analytical chemistry. Here, we thoroughly characterized the influence of MS and LC settings — of a Q Exactive HF operated in Full MS/data-dependent MS^2^ TOP *N* acquisition mode — in order to optimize the semi-quantification of polar lipids. Optimization of MS-source settings improved the signal intensity by factor 3 compared to default settings. Polar lipids were separated on an ACQUITY Premier CSH C18 reversed-phase column (100 × 2.1 mm, 1.7 µm, 130 Å) during an elution window of 28 min, leading to a sufficient number of both data points across the chromatographic peaks, as well as MS^2^ spectra. Analysis was carried out in positive and negative ionization mode enabling the detection of a broader spectrum of lipids and to support the structural characterization of lipids. Optimal sample preparation of biological samples was achieved by liquid–liquid extraction using MeOH/MTBE resulting in an excellent extraction recovery > 85% with an intra-day and inter-day variability < 15%. The optimized method was applied on the investigation of changes in the phospholipid pattern in plasma from human subjects supplemented with n3-PUFA (20:5 and 22:6). The strongest increase was observed for lipids bearing 20:5, while 22:4 bearing lipids were lowered. Specifically, LPC 20:5_0:0 and PC 16:0_20:5 were found to be strongest elevated, while PE 18:0_22:4 and PC 18:2_18:2 were decreased by n3-PUFA supplementation. These results were confirmed by targeted LC–MS/MS using commercially available phospholipids as standards.

**Graphical abstract:**

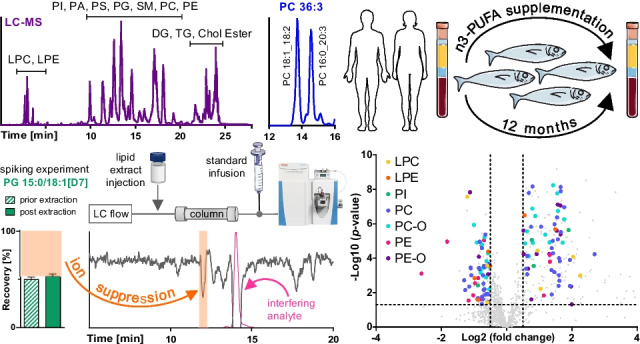

**Supplementary information:**

The online version contains supplementary material available at 10.1007/s00216-023-05080-0.

## Introduction

Lipids are lipophilic small molecules involved in many biological functions including cell membrane assembly, energy metabolism, cell signaling, and regulation of inflammation. They are classified in eight major lipid categories: fatty acids, sphingolipids, glycerolipids, glycerophospholipids, sterol lipids, prenol lipids, saccharolipids, and polyketides [1]. Fatty acids can be saturated, e.g., stearic acid (18:0), monounsaturated, or polyunsaturated of the n3, n6, or n9 series, e.g., eicosapentaenoic acid (EPA, 20:5(5Z,8Z,11Z,14Z,17Z)), arachidonic acid (ARA, 20:4(5Z,8Z,11Z,14Z)), or oleic acid (18:1(9Z)) [2]. Fatty acids are the major components of phospholipids which are the main constituents of the cellular membrane [3, 4]. Driven by the biological importance, lipidomics has emerged as a major field of research in life sciences in the past decades. However, due to the structural diversity of lipids, a simultaneous analysis of “all”, i.e., a comprehensive set of lipids in untargeted lipidomics is challenging.

Compared to shotgun lipidomics, liquid chromatography coupled to mass spectrometry (LC–MS) allows to separate overlapping isomeric and isobaric lipid species in complex biological matrices [5]. The separation of lipids can be carried out by normal-phase liquid chromatography (NPLC) and hydrophilic interaction liquid chromatography (HILIC) allowing the separation based on the lipid classes, e.g., by the head group of phospholipids, or by reversed-phase liquid chromatography (RPLC) where the separation of the lipids is based on their hydrophobicity, i.e., the length of the fatty acyl chains and degree of unsaturation [6]. RPLC comprising about 71% of all lipidomics applications has been most widely used for the analysis of complex lipids using C8 [7], C18 [8, 9], or C30 [10] modified silica columns. Criscuolo *et al. *showed that a C18 column achieved a better separation for polar lipids such as lysophospholipids, sphingomyelin, or glycerophospholipids compared to a C30 column [11]. Separation of isobaric phospholipid species was previously achieved using C18 columns, e.g., ACQUITY UPLC BEH C18 [12] or ACQUITY Premier CSH C18 [13].

For MS detection, untargeted analysis using high resolution and acquisition in Full MS mode is a promising approach as it allows simultaneous and comprehensive monitoring of a broad range of lipids extracted from a biological sample. Additionally, using data-dependent fragmentation, product ion spectra are obtained helping to identify the individual lipid species based on distinct fragmentation behavior. The processing of this huge amount of data generated during untargeted analysis is performed with bioinformatic software in two steps including first peak detection and peak alignment, and subsequent lipid annotation using comprehensive databases [6, 14]. Different software packages for the processing of lipid data have been developed and are available open source, as well as commercially [15].

Targeted LC–MS/MS analysis only allows the detection of preselected analytes of interest. During method development, a focus is set on the optimization of LC and MS parameters, assuring an optimal specific and sensitive detection of these analytes, e.g., eicosanoids and other oxylipins [16] or peptides [17]. Among the large number of LC-high-resolution-MS (LC-HRMS) methods which have been described for the analysis of lipids in biological samples such as plasma [8, 11], serum [18], or liver [10], only few characterize and optimize the instrumental parameters including source parameters for ionization efficiency as well as parameter settings for Full MS and data-dependent acquisition. For example, Narváez-Rivas *et al.* optimized the MS parameters of the Q Exactive HF for the analysis of phospholipids in rat plasma but provided no information about the effects of the parameters [10].

Overall, the main focus of method development/characterization of current lipidomics approaches is set on the bioinformatic processing of the LC–MS data. For example, the performance of extraction procedures [19, 20] and chromatographic separation [8, 11] is usually evaluated based on the number of identified lipid species by the bioinformatic software. However, characterization of ionization efficiency and extraction yield of representative lipid species as it is carried out in targeted LC–MS/MS analysis is limited and only described in few untargeted approaches [21].

The aim of this work was developing and optimizing extraction, separation, and detection of an untargeted LC-HRMS method for the identification and semi-quantitative analysis of lipids with a focus on phospholipids. The careful optimization of the MS parameters allowed to improve the ionization efficiency of the phospholipids. The optimization of the chromatographic separation on two different columns aimed to achieve a broad elution range for phospholipids allowing to detect more analytes by Full MS/ddMS^2^ acquisition. Combined with the ionization in both modes, it enabled to acquire a broader spectrum of lipids and to confirm the characterization of tentatively identified lipids. Semi-quantification of polar lipids was performed using one internal standard (IS) per lipid class. Two liquid–liquid extraction protocols were compared and evaluated regarding extraction efficiency of IS and intra- and inter-day variability. Matrix effects were investigated by ion suppression analysis using two different pools of plasma, unveiling ion suppression as well as ion enhancement effects. Finally, the method was applied to investigate the effects of n3-polyunsaturated fatty acid (PUFA) supplementation on the human plasma lipidome. Here, we could identify distinct phospholipids which were increased and decreased by 12 months of n3-PUFA supplementation.

## Material and methods

### Chemicals

SPLASH Lipidomix Mass Spec Standard mixture containing deuterium-labeled lipids from 14 different lipid classes (lysophosphatidylcholine (LPC) 18:1[D7]/0:0, lysophosphatidylethanolamine (LPE) 18:1[D7]/0:0, monoacylglycerol (MG) 18:1[D7]/0:0/0:0, phosphatidylserine (PS) 15:0/18:1[D7], phosphatidylinositol (PI) 15:0/18:1[D7], phosphatidylglycerol (PG) 15:0/18:1[D7], phosphatidic acid (PA) 15:0/18:1[D7], sphingomyelin (SM) 18:1;2O/18:1[D7], phosphatidylcholine (PC) 15:0/18:1[D7], phosphatidylethanolamine (PE) 15:0/18:1[D7], diacylglycerol (DG) 15:0/18:1[D7]/0:0, triacylglycerol (TG) 15:0/18:1[D7]/15:0, cholesteryl ester (Chol Ester) 18:1[D7] and free cholesterol (FC) [D7]; 550 nM–5 µM; concentrations relative to ratios in human plasma, for individual concentrations of the standards in the plasma extract see Table 1) and phospholipid standards PC 16:0/20:4(5Z,8Z,11Z,14Z), PE 18:1(9Z)/18:1(9Z), and PE 14:0/14:0 were purchased from Avanti Polar Lipids (local supplier: Merck KGaA, Darmstadt). PC 18:2(9Z,12Z)/18:2(9Z,12Z), PC 18:0/20:5(5Z,8Z,11Z,14Z,17Z), PC 18:0/22:6(4Z,7Z,10Z,13Z,16Z,19Z), PE 18:0/20:4(5Z,8Z,11Z,14Z), and PE 18:0/22:4(7Z,10Z,13Z,16Z) used for targeted LC–MS/MS were from Cayman Chemical (local supplier: Biomol, Hamburg, Germany).

**Table 1:** Characterization of mass spectrometric and chromatographic parameters of deuterium-labeled lipids used as IS. Shown are the molecular formula, the mass-to-charge ratio (*m/z*) of the most intense adduct measured in ESI(+) and ESI(-) mode, the concentration of the IS in the plasma extract, the retention time (*t*_R_), and the full width at half maximum (FWHM) of the chromatographic peak using the LC-HRMS method

Analyte	Molecular formula	ESI(-)		ESI( +)		Concentration [µM]	*t*_*R*_ ± SD [min] (rel. SD)^1^	FWHM^1^ ± SD [s]
		*m/z*	Adduct type	*m/z*	Adduct type			
LPC 18:1[D7]/0:0	C_26_H_45_D_7_NO_7_P	573.3903	[M+COOH]^−^	529.3994	[M+H]^+^	4.5	2.88 ± 0.01 (0.31%)	3.98 ± 0.46
LPE 18:1[D7]/0:0	C_23_H_39_D_7_NO_7_P	485.3379	[M-H]^−^	487.3524	[M+H]^+^	1.0	2.97 ± 0.01 (0.32%)	4.15 ± 0.65
PI 15:0/18:1[D7]	C_42_H_72_D_7_O_13_P	828.5625	[M-H]^−^	847.6036	[M+NH_4_]^+^	1.0	10.58 ± 0.04 (0.38%)	10.04 ± 0.48
PS 15:0/18:1[D7]	C_39_H_67_D_7_NO_10_P	753.5417	[M-H]^−^	755.5562	[M+H]^+^	0.50	11.13 ± 0.08 (0.71%)	12.55 ± 1.02
PG 15:0/18:1[D7]	C_39_H_68_D_7_O_10_P	740.5464	[M-H]^−^	759.5875	[M+NH_4_]^+^	3.5	11.43 ± 0.05 (0.42%)	10.75 ± 1.19
SM 18:1;2O/18:1[D9]	C_41_H_72_D_9_N_2_O_6_P	782.6379	[M+COOH]^−^	738.6470	[M+H]^+^	4.0	12.77 ± 0.05 (0.38%)	11.92 ± 1.00
PC 15:0/18:1[D7]	C_41_H_73_D_7_NO_8_P	797.6049	[M+COOH]^−^	753.6134	[M+H]^+^	20	14.89 ± 0.05 (0.35%)	12.25 ± 1.5
PE 15:0/18:1[D7]	C_38_H_67_D_7_NO_8_P	709.5519	[M-H]^−^	711.5664	[M+H]^+^	0.75	16.17 ± 0.05 (0.33%)	12.33 ± 0.39
DG 15:0/18:1[D7]/0:0	C_36_H_61_D_7_O_5_	^2^	-	605.5844	[M+NH_4_]^+^	1.5	22.42 ± 0.02 (0.08%)	3.98 ± 0.53
TG 15:0/18:1[D7]/15:0	C_51_H_89_D_7_O_6_	^2^	-	829.7985	[M+NH_4_]^+^	6.5	23.97 ± 0.01 (0.03%)	3.60 ± 0.36
Chol Ester 18:1[D7]	C_45_H_71_D_7_O_2_	^2^	-	675.6779	[M+NH_4_]^+^	50	24.35 ± 0.01 (0.04%)	3.87 ± 0.47

^1^*t*_R_ and FWHM were determined as mean (± standard deviation (SD)) in plasma extracts which were pre-spiked with the IS mixture and measured on 6 different days (*n* = 92)

^2^DG 15:0/18:1[D7]/0:0, TG 15:0/18:1[D7]/15:0, and Chol Ester 18:1[D7] are only detected in ESI(+) mode

Acetonitrile (ACN) LC–MS grade, methanol (MeOH) LC–MS grade, *iso-*propanol (IPA) LC–MS grade, chloroform (CHCl_3_) HPLC grade, formic acid LC–MS grade, and *n-*hexane HPLC grade were obtained from Fisher Scientific (Schwerte, Germany). Ultra-pure water (18.2 MΩ) was generated using the Barnstead Genpure Pro system from Thermo Fisher Scientific (Langenselbold, Germany). Ammonium formate was supplied by Sigma-Aldrich (Schnelldorf, Germany). All other chemicals, including *tert*-butyl methyl ether (MTBE), were purchased from Merck KGaA (Darmstadt, Germany).

For method characterization, 3 different pools of human plasma were generated. The blood was collected from healthy human subjects in accordance with the guidelines of the Declaration of Helsinki and approved by the ethics committee of the University of Wuppertal. The blood was collected in EDTA tubes and centrifuged (4 °C, 10 min, 1200 × *g*). The plasma was collected, aliquoted, and stored at − 80 °C as described in [22].

### Lipid extraction

#### Extraction using MeOH and MTBE

Lipids in plasma were extracted using a modified liquid–liquid extraction (LLE) based on Matyash *et al.* [23, 24]. Briefly, 10 µL freshly thawed plasma was transferred to glass tubes followed by the addition of 10 µL SPLASH internal standards (IS). 225 µL MeOH was added and samples were vortexed shortly. Then, 750 µL MTBE was added and samples were thoroughly vortexed for 2 min. Phase separation was induced by addition of 188 µL 150 mM ammonium acetate and centrifugation (4 °C, 10 min, 1000 × *g*). The upper organic phase was carefully collected in glass tubes containing 6 µL 30% glycerol in MeOH and the lower phase was re-extracted by addition of 300 µL MTBE. The samples were vortexed for 1 min and centrifuged again (4 °C, 5 min, 1000 × *g*). The combined upper phases were evaporated to dryness using a vacuum concentrator (1 mbar, 30 °C, ∼70 min; Christ, Osterode am Harz, Germany). The residue was reconstituted in 50 μL of 50/50 mobile phase (A/B, *v/v* without modifier, i.e., IPA/ACN/H_2_O (45/35/20, *v/v/v*)) containing 200 nM PE 14:0/14:0 as IS2. Samples were sonicated, centrifuged, and transferred to vials for LC-HRMS analysis.

#### Extraction using IPA, *n*-hexane, CHCl_3_, and MeOH [25]

Lipids in plasma were extracted using a two-stepped LLE [26, 27]. 10 µL freshly thawed plasma was transferred to glass tubes followed by the addition of 10 µL IS (SPLASH) and 185 µL water. After addition of 1 µL glacial acetic acid, the phase separation was induced with 500 µL 1 M acetic acid/IPA/*n*-hexane (2/20/30, *v/v/v*) and samples were vortexed for 1 min. 500 µL *n*-hexane was then added, samples were vortexed again for 1 min and centrifuged (room temperature, 10 min, 1000 × *g*). The upper phase was carefully collected in glass tubes containing 6 µL 30% glycerol in MeOH. The lower phase was washed with 500 µL *n*-hexane, vortexed for 1 min, and centrifuged again (room temperature, 10 min, 1000 × *g*) and the upper layers were combined. In the second step of the extraction, 750 µL CHCl_3_/MeOH (1/2, *v/v*) was added to the lower aqueous phase and samples were vortexed for 1 min. 250 µL CHCl_3_ was further added and samples were vortexed again for 1 min. Phase separation was induced by addition of 250 µL 150 mM ammonium acetate and centrifugation (room temperature, 10 min, 1000 × *g*). The upper phase was discarded and the lower phase was collected and combined with the upper phases from the first extraction step. The combined organic phases containing the lipids of both extraction steps were evaporated to dryness using a vacuum concentrator (1 mbar, 30 °C, ∼70 min). The residue was reconstituted in 50 μL IPA/ACN/H_2_O (45/35/20, *v/v/v*) containing 200 nM PE 14:0/14:0 as IS2, sonicated, centrifuged, and transferred to vials for LC-HRMS analysis.

### Method characterization

Two established LLE protocols used in the lipidomics field were tested and compared regarding their extraction recovery of major lipid classes from plasma: a protocol using MeOH/MTBE based on a modified procedure according to Matyash *et al.* [23], and a two-stepped extraction using acetic acid/IPA*/n*-hexane based on Hara *et al.* [27] for the first step and CHCl_3_/MeOH based on Bligh and Dyer [26] for the second [25].

Extraction efficiency was evaluated for both protocols on three different days by determining the recovery of the IS spiked to the sample prior to extraction normalized to the IS2 (i.e., PE 14:0/14:0), which was added in the last step before LC-HRMS analysis. The recovery was calculated relative to the recovery of an IS solution directly injected.

Further lipid extraction was carried out using MeOH/MTBE. Robustness of the extraction was assessed by three different operators on three different days using three different pools of plasma. Effects of matrix were investigated by assessing the extraction recovery of IS added to plasma prior to or post extraction and also using different volumes of plasma for extraction. Furthermore, matrix effects were determined by ion suppression analysis by post-columnly infusing a diluted (0.37–10 µM) SPLASH solution (5 µL/min) mixed *via* a T-piece with the LC flow (260 µL/min) after injection of a plasma extract without IS (Figs. 3 and 4).

### Untargeted LC-HRMS instrument method

Untargeted lipidomics analysis was performed on a Vanquish Horizon ultra-high-performance liquid chromatography system composed of an autosampler, a binary pump, and a column oven coupled to a hybrid quadrupole-orbitrap mass spectrometer (Q Exactive HF) (Thermo Fisher Scientific, Dreieich, Germany). Samples (5 µL) were injected into the LC-HRMS system using an autosampler equipped with a 25-µL sample loop. The sample rack was kept at 10 °C. Two columns were tested for the separation, i.e., ZORBAX Eclipse Plus RRHT C18 (2.1 × 150 mm, 1.8 µm, 95 Å; Agilent, Waldbronn, Germany) and ACQUITY Premier CSH C18 (2.1 × 100 mm, 1.7 µm, 130 Å; Waters, Eschborn, Germany). A binary gradient was used with eluent A (H_2_O/ACN, 40/60, *v/v*) and eluent B (IPA/ACN, 90/10, *v/v*, 1% H_2_O), both containing 10 mM ammonium formate and 0.1% formic acid. The optimized chromatographic separation was carried out on the ACQUITY Premier CSH C18 column equipped with a guard column (2.1 × 5 mm, 1.7 µm) at 40 °C using the following gradient with a flow rate of 260 µL/min: 0–0.7 min 30% B; 0.7–0.8 min 30–52.5% B; 0.8–11 min 52.5% B; 11–20 min 52.5–60% B; 20–22 min 60–99% B; 22–26 min 99% B; 26–28 min 30% B for column washing and re-equilibration. The total analysis time was 28 min.

**Fig. 1:**
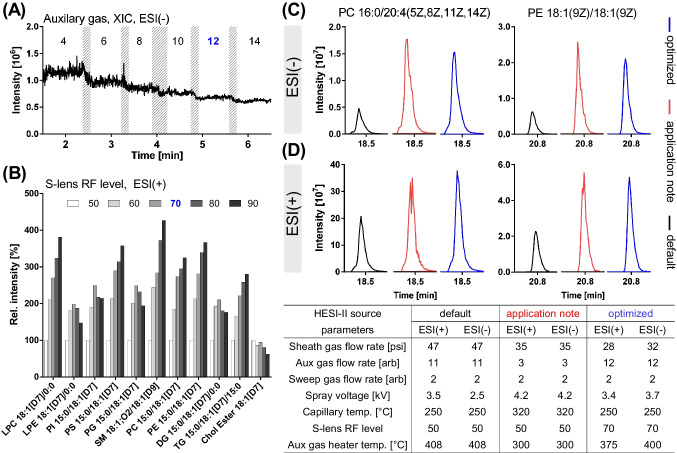
Effect of selected ion source parameters on intensity and stability of the ESI–MS signal. Shown is **A** the influence of the auxiliary gas flow rate on the signal in ESI(-) mode during infusion of a phospholipid standard containing PC 16:0/20:4(5Z,8Z,11Z,14Z) and PE 18:1(9Z)/18:1(9Z) while other parameters were set to default; **B** the effect of the S-lens RF level on signal intensity relative to a value of 50 for different deuterium-labeled lipids; **C**, **D** peak intensity and shape of PC 16:0/20:4(5Z,8Z,11Z,14Z) and PE 18:1(9Z)/18:1(9Z) in (*top*, **C**) ESI(-) and (*bottom*, **D**) ESI(+) mode using default parameters, parameters of an application note for lipidomics from the manufacturer [33], or parameters after optimization

Lipids were analyzed following positive and negative electrospray ionization in two separate runs using a heated electrospray ionization (HESI) source. For optimization of ionization parameters, PC 16:0/20:4(5Z,8Z,11Z,14Z) and PE 18:1(9Z)/18:1(9Z) were chosen as representative lipids for two abundant phospholipid classes found in plasma. Optimization was done in positive and negative mode by infusion of the standards (each 300 nM) (Fig. 1) *via* a syringe pump with 5 µL/min combined *via* a T-piece with an LC flow of 260 µL/min at an eluent composition of 70% B. Alternatively, also flow injection analysis injecting 5 µL of the standards in the LC flow (i.e., 260 µL/min) was used. Optimized settings are summarized in Fig. 1. These standards were also used for the optimization of the normalized collision energy (NCE) (Fig. S2).

As sheath gas, auxiliary gas, sweep gas, and collision gas nitrogen was used, generated from compressed air further purified with the purifier RAMS05Z, and combined with the NGM33 nitrogen generator (CMC instruments, Eschborn, Germany). The offset of the sprayer was side-to-side + 1, front-to-back 1.75 μm, and depth between C- and D-ring.

MS detection was carried out in Full MS data-dependent (dd) MS^2^ TOP *N* mode (Full MS/ddMS^2^). For Full MS scans, data were acquired over a mass range of *m/z* 200–1200, for both positive and negative ionization mode. The Full MS scans were recorded at a resolution setting of 60,000 with the automatic gain control (AGC) target set to 1 × 10^6^ and a maximum ion injection time (IT) of 160 ms. Data-dependent scans from the TOP *N* *m/z* detected in the Full MS scans were triggered based on an exclusion and inclusion list specific for positive and negative ionization (Table S1 and S2) and a minimum AGC target of 2 × 10^3^ considering an apex trigger of 1–4 s and a dynamic exclusion time of 4 s. MS^2^ scans were acquired from the triggered *m/z* with an isolation window of *m/z* 1.5 using NCE combining 20 and 25 relative to *m/z* 500, at a resolution setting of 15,000 with an AGC target of 5 × 10^4^ and a maximum IT of 80 ms. In order to ensure enough data points across the chromatographic peaks in Full MS mode for semi-quantitative evaluation, the analysis time was split into segments with different numbers of MS^2^ scans triggered during one duty cycle: between 0 and 7 min TOP 5, between 7 and 12 min TOP 10, and between 12 and 28 min TOP 15.

Mass accuracy was assured using the following lock masses at the beginning of the run: in positive mode *m/z* 391.2843 (polytetrafluoroethylene) between 1.1 and 1.2 min and in negative mode *m/z* 265.1479 (sodium dodecyl sulfate) between 1.55 and 1.65 min. Mass calibration was carried out every 72 h by infusion of Pierce LTQ Velos ESI Positive Ion Calibration Solution and Pierce ESI Negative Ion Calibration Solution (Thermo Fisher Scientific, Langenselbold, Germany).

For data acquisition and instrument control Chromeleon software (version 7.2.11, Thermo Fisher Scientific) was used.

### Targeted LC–MS/MS instrument method

Targeted LC–MS/MS analysis of selected phospholipids was carried out using a 1290 Infinity II (Agilent, Waldbronn, Germany) LC system composed of an autosampler, a binary pump, and a column oven. The separation of the lipids was achieved using the same chromatographic conditions described for the untargeted LC-HRMS method. The LC system was coupled to a QTRAP 6500+ mass spectrometer (Sciex, Darmstadt, Germany) operated in negative electrospray ionization mode with the following settings: ion spray voltage − 4500 V, source temperature 650 °C, nebulizer gas (GS1, compressed air purified with RAMS05Z; CMC instruments, Eschborn, Germany) 60 psi and drying gas (GS2, purified compressed air) 60 psi, curtain gas (nitrogen, generated with the nitrogen generator Eco Inert-ESP4; DWT, Bottrop, Germany) 35 psi, collision gas (nitrogen) 6 psi. MS detection was carried out in scheduled multiple reaction monitoring (MRM) mode acquiring two transitions per phospholipid: one for quantification and one for qualification resulting from the cleavage of the individual fatty acyl chains. The detection window was set to 180 s around the retention time and the cycle time to 0.4 s. Declustering potentials (DP), entrance potentials (EP), collision cell exit potentials (CXP), and collision energies (CE) were optimized for each of the phospholipids and transitions using flow injection analysis with standards (Fig. S7). MS parameters for targeted phospholipid analysis can be found in Table S4. Analyst (Sciex, version 1.7) was used for instrument control and data acquisition, and Multiquant (Sciex, version 2.1.1) for data evaluation.

For calibration, stock solutions of the individual phospholipids PC 18:2(9Z,12Z)/18:2(9Z,12Z), PC 18:0/20:5(5Z,8Z,11Z,14Z,17Z), PC 18:0/22:6(4Z,7Z,10Z,13Z,16Z,19Z), PE 18:0/20:4(5Z,8Z,11Z,14Z), and PE 18:0/22:4(7Z,10Z,13Z,16Z) were mixed and diluted in glass volumetric flasks (5 mL) with ACN/IPA (50/50, *v/v*) at 9 concentration levels. Each calibration level contained the same amount of the IS (SPLASH) comprising one labeled phospholipid from each lipid class (400 nM for PC 15:0/18:1[D7] and 15 nM for PE 15:0/18:1[D7]). Calibration curves were calculated using linear least square regression (weighting: 1/*x*^2^). Analyte quantification was carried out based on the analyte to corresponding IS peak area ratio using the obtained calibration curves. Linearity was assessed using standard solutions covering a concentration range from 0.2 to 1000 nM. The limit of detection (LOD) was determined as the concentration yielding a signal-to-noise ratio (*S*/*N*, peak to peak) ≥ 3. The concentration with a *S*/*N* ≥ 5 and an accuracy of 80–120% within the calibration curve was defined as lower limit of quantification (LLOQ) and set as the lowest concentration of the calibration curve (Table S4).

### n3-PUFA supplementation study

The effects of n3-PUFA supplementation on the lipid pattern were investigated in plasma samples derived from a double-blinded, randomized, controlled intervention trial [28]. A subset of human plasma samples from 20 participants (9 males, 11 females, age 23–72 years) out of 42 subjects who received n3-PUFA capsules containing EPA and DHA corresponding to 4 portions of fatty fish per week (1.5 g EPA and 1.77 g DHA as triacylglycerols per portion) was selected. Criteria for the inclusion/exclusion of subjects are summarized in Figure S8. Plasma samples of the individual participants at baseline (before supplementation) and after 12 months of supplementation were extracted using the LLE with MeOH/MTBE and analyzed by the untargeted LC-HRMS method in randomized order.

### Data processing

Raw data acquired in positive and negative ionization mode by untargeted LC-HRMS analysis were processed using MS-DIAL software (version 4.70) [29] for feature detection, spectra deconvolution, and peak alignment between samples. Parameter settings for data processing by MS-DIAL are summarized in Table S3. Each detected feature was manually reviewed and assigned to a putative lipid class and to one of the three different categories (i.e., Confidence, Unsettled, or Unknown) based on its retention time and fragmentation spectrum according to following criteria: lipid class assignment was done based on following plausible retention times: lysophospholipids (≤ 8 min), glycerophospholipids (≥ 4 min), SM (≥ 4 min), DG (≥ 12 min), and TG (≥ 15 min). Features assigned to one of the mentioned lipid classes with a retention time outside the defined range were flagged as “Unknown” and were not further evaluated. An “Unsettled” feature with an identification score between 70 and 75% was flagged as “Confidence” if its fragmentation spectrum contained in positive mode (i) the precursor ion, (ii) a fragment of a neutral loss of a fatty acyl (e.g., ketene), and (iii) characteristic fragments of the lipid class (e.g., *m/z* 184.0733 for PC) [30]. In negative mode, a feature was assigned to “Confidence” if its fragmentation spectrum contained (i) the fragment(s) of the fatty acyl(s), (ii) a fragment of a neutral loss of a fatty acyl (e.g., ketene), and (iii) characteristic fragments of the lipid class (e.g., *m/z* 168.0426 for PC) [30]. In case one of the criteria mentioned above was not fulfilled, the feature remained assigned “Unsettled”. Likewise, features having an identification score between 67 and 70% were rated as “Unsettled”. All features assigned to “Confidence” and “Unsettled” were included in the further data evaluation. For additional confidence of lipid identification, the retention time of a logical series of lipid species from the same lipid class was plotted against the sum of the fatty acyl chain length or the sum of the number of double bonds according to Vaňková *et al.* [12] (Fig. S9-10).

The peak heights of features detected in positive and/or negative mode derived from MS-DIAL data evaluation were normalized to peak heights of the IS from the same lipid class. For the individual features, fold changes between baseline (before supplementation) and after 12 months of supplementation were calculated for individual participants and the mean fold changes were determined. Using *t*-test, for the individual features, *p*-values for the means of the normalized peak heights before and after supplementation were determined. Volcano plots were created by plotting the log2 (fold change) against the − log10 (*p*-value).

Putatively identified phospholipids, which changed most after n3-PUFA supplementation and were commercially available, were quantified by targeted LC–MS/MS using the same plasma extracts diluted 1:50 in ACN/IPA (50/50, *v/v*).

### Lipid notation

The shorthand notation of the lipids is based on Liebisch* et al*. using the separator “_” if the *sn*-position of the fatty acyl(s) chain is unknown, while the separator “/” indicates a proven *sn*-position (with *sn*-1/*sn*-2) [31]. The position of the double bonds, e.g., PC 16:0/20:4(5Z,8Z,11Z,14Z), was specified only if confirmed by authentic standards.

## Results and discussion

### Optimization of mass spectrometric parameters

Sensitive as well as selective mass spectrometric analysis of phospholipids in biological samples requires careful optimization of instrument parameters. Mass spectrometric detection of phospholipids is feasible after positive (ESI(+)) and negative (ESI(-)) electrospray ionization due to their polar head group by forming different types of adducts [6], while DG, TG, and Chol Ester can only be efficiently ionized in positive mode [8, 32]. Analysis of lipids was carried out in positive and negative mode in two separate runs to enable characterization of the lipid class as well as the fatty acyl chains based on characteristic fragmentation behavior. The instrument software sets default settings for the source parameters based on the LC flow rate. Starting from these settings, the source parameters of the HESI source (i.e., spray voltage, sheath gas, auxiliary gas, auxiliary gas heater temperature, sweep gas, capillary temperature) and the S-lens RF level were optimized in both ionization modes, and compared with the default settings and the settings described in an application note for lipidomics analysis with the same flow rate (260 µL/min) from the instrument manufacturer [33]. The optimization of the spray voltage, the sheath gas, the sweep gas, and the capillary temperature showed only minor impact on the signal intensity, and is described in detail in the supplementary information (Fig. S1).

The heated auxiliary gas flow was optimized in combination with the auxiliary gas heater temperature and showed the greatest impact among all source parameters on spray stability in both ionization modes. It was optimized in a range of 4–14 arbitrary units (Fig. 1A). A value of 4 resulted in a noisy and unstable spray, and increasing the auxiliary gas flow decreased the noise but also decreased the signal intensity. A value of 12 was selected for both ionization modes in order to minimize the noise of the spray without losing too much sensitivity. The value selected for the auxiliary gas flow is close to the default settings (i.e., 11), but clearly higher than the value used in the application note (i.e., 3) [33]. However, the peak shape of the phospholipids is clearly improved when a higher value is used (Fig. 1C, D). Our results are in line with previous studies employing with the same instrument an auxiliary gas flow of 10 for the analysis of phospholipids in serum at an LC flow rate of 400 µL/min [34], or in pituitary adenoma tissues at an LC flow rate of 260 µL/min [7]; or an auxiliary gas flow of 15 for the analysis of human plasma lipids at an LC flow rate of 325 µL/min [11].

Transfer of ions from the ion transfer tube to the ion optics through the S-lens is achieved by the RF amplitude applied to the electrodes of the S-lens. The S-lens RF level is a numerical factor affecting transmission, i.e., a higher S-lens RF level increases transmission of ions with higher *m/z* while also fragmentation of fragile ions in the S-lens occurs. Optimization of the S-lens RF level was performed using deuterium-labeled lipids (SPLASH) from different lipid classes. Increasing the S-lens RF level from the default value, i.e., 50, increased the ion transmission of all IS except Chol Ester 18:1[D7]. For a S-lens RF level of 80 and 90, a decrease in the signal of LPE 18:1[D7]/0:0, PI 15:0/18:1[D7], PG 15:0/18:1[D7], and DG 15:0/18:1[D7]/0:0 was observed. Thus, a S-lens RF level of 70 for both ionization modes was chosen which massively improved the ion transmission of the phospholipids (Fig. 1B). The default S-lens RF level of 50 is used in several lipidomic methods using a Q Exactive HF instrument [7, 9, 35]. However, our results show that optimization of this parameter has a great impact on the ion transmission and signal intensity and should thus be carefully optimized.

Overall, the optimization of the source parameters minimized the noise resulting in a better peak shape, and increased the sensitivity by a factor of 3 in ESI(+) and ESI(-) compared to the default parameters.

### Optimization of chromatographic separation

Liquid chromatographic separation of lipids covering the range from polar lipids, such as lysophospholipids, to the very hydrophobic ones, e.g., neutral lipids, can be performed using RPLC [5, 8, 34, 36]. We aimed to optimize the chromatographic conditions in order to achieve an efficient separation between different lipid species with a focus on phospholipids. Since numerous isobaric lipid species, i.e., with the same *m/z* are present in biological samples, liquid chromatographic separation is crucial for their characterization. An isocratic step was included in the gradient to extend the elution window for phospholipids, thereby enabling the acquisition of more MS^2^ spectra for their characterization in Full MS/ddMS^2^ mode.

For the liquid chromatographic optimization, we selected three critical pairs of isobaric phospholipids: PC 18:2_20:4/PC 18:1_20:5, PC 18:1_18:2/PC 16:0_20:3, and PC 18:0_18:2/PC 18:1_18:1. These are highly abundant in human plasma and have different retention times between 10 and 20 min under the applied RPLC conditions. Two RP C18 columns with fully porous sub-2-µm particles for high separation efficiency and sample loading capacity were tested using a lipid extract from human plasma. We chose two columns which were previously successfully used in lipidomics applications, i.e., the ZORBAX Eclipse Plus RRHT C18 [37, 38] and the ACQUITY Premier CSH C18 column [7, 9, 34, 38, 39].

**Fig. 2:**
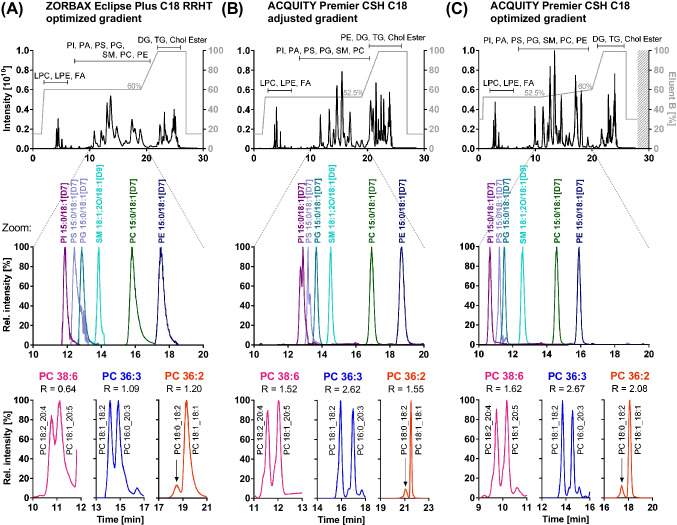
Chromatographic separation efficiency of lipids. Shown is the separation (*top*) of a lipid extract from human plasma (Full MS scan *m/z* 200–1200), (*middle*) of deuterium-labeled IS (respective XIC), and (*bottom*) of isobaric phospholipid species PC 38:6 (*m/z* 806.5694), PC 36:3 (*m/z* 784.5851), and PC 36:2 (*m/z* 786.6007) acquired in ESI(+) mode using **A** an optimized gradient on a ZORBAX Eclipse Plus RRHT C18 column (2.1 × 150 mm, 1.8 µm, 95 Å), **B** an ACQUITY Premier CSH C18 column (2.1 × 100 mm, 1.7 µm, 130 Å) and a gradient with adjusted elution power, and **C** an optimized gradient on the latter column. Mobile phases for all separations were eluent A (H_2_O/ACN (40/60, *v/v*)) and eluent B (IPA/ACN (90/10, *v/v*), 1% H_2_O), both containing 10 mM ammonium formate and 0.1% formic acid. The fatty acyl chains of the isobaric phospholipids (bottom) were characterized based on the fragmentation spectra acquired in ESI(-) mode

Using the ZORBAX Eclipse Plus RRHT C18 column (2.1 × 150 mm, 1.8 µm, 95 Å) with an optimized gradient, lipids from a human plasma extract eluted as relatively broad peaks with a full width at half maximum (FWHM) for the labeled IS PS 15:0/18:1[D7], PC 15:0/18:1[D7], and PE 15:0/18:1[D7] of 17.4 s, 19.8 s, and 18.9 s, respectively. Phospholipids showed also an asymmetric peak shape with a tailing factor for the labeled phospholipids between 1.62 and 4.38. Overall, this mediocre separation of the lipids is reflected by incomplete separation of the selected critical pairs, i.e., PC 18:2_20:4/PC 18:1_20:5 and PC 18:1_18:2/PC 16:0_20:3 with a resolution of 0.64 and 1.09, respectively. Additionally, the pair PC 18:0_18:2/PC 18:1_18:1 was not fully separated (*R* = 1.20) (Fig. 2A).

The separation on the ACQUITY Premier CSH C18 column (2.1 × 100 mm, 1.7 µm, 130 Å) was carried out with adjusted elution power of the gradient during the isocratic step by lowering the percentage of eluent B from 60 to 52.5% (Fig. 2B). With this column, the same elution order was observed; however, the peak shape of the lipids was considerably improved showing narrower peaks and better peak symmetry. No peak tailing was observed for the phospholipids, except for the acidic PS 15:0/18:1[D7] (i.e., tailing factor 1.81) (Fig. 2B, middle), which is known in RPLC even when a high aqueous percentage is used for the initial conditions [40]. The better performance of this column might be explained by the charged surface of its particles improving peak symmetry with low ionic-strength mobile phases.

Separation of phospholipids was also considerably improved with a resolution ≥ 1.5 for the selected critical pairs, e.g., PC 18:2_20:4/PC 18:1_20:5 (*R* = 1.52) (Fig. 2B, bottom). However, using this gradient (Fig. 2B), late-eluting PC and PE species still showed broad peaks, e.g., PE 15:0/18:1[D7] eluting at the end of the isocratic step with a FWHM of 16.8 s. Also, more hydrophobic phospholipids eluting after the isocratic step, e.g., PE 18:0_20:4 at 21.7 min or PC 18:0_20:3 at 21.9 min, co-eluted with other late-eluting phospholipids as indicated in the TIC (Fig. 2B, top). Thus, the gradient was further optimized by including a shallow linear increase to 60% B after the isocratic step and adjusting the initial percentage of B as well as the time for the final elution and re-equilibration step: The more hydrophobic phospholipids eluted earlier, e.g., PE 18:0_20:4 at 18.6 min and PC 18:0_20:3 at 19.7 min, and their separation was improved. For the starting conditions, 30% B was used as isocratic pre-concentration step to focus the analytes at the beginning of the column. With a capacity factor *k* > 1, the retention of the polar LPC 18:1[D7]/0:0 (*k* = 2.71) was sufficient.

Overall, with this optimized gradient, the peak shape was further improved yielding FWHM for all labeled phospholipids between 10 and 13 s, also for PE 15:0/18:1[D7] (Table 1). Moreover, separation of the critical pairs was optimal with a resolution ≥ 1.5, i.e., PC 18:2_20:4/PC 18:1_20:5 (*R* = 1.62), PC 18:1_18:2/PC 16:0_20:3 (*R* = 2.67), and PC 18:0_18:2/PC 18:1_18:1 (*R* = 2.08) (Fig. 2C, bottom). The isocratic step at 99% B at the end of the gradient was held for 4 min, which was sufficient to elute the neutral lipids, i.e., TG, DG, and Chol Ester (leading to minimal carry-over for TG 18:1_18:1_18:1 to the next injection (< 1%)). Only 2-min re-equilibration was required, resulting in stable retention times for the lysophospholipids in the following injection. Including re-equilibration, the final run time of the optimized method was 28 min covering polar as well as neutral lipids and the method showed stable retention times with an inter-batch (*n* = 92, 6 days) relative standard deviation (RSD) < 0.71% (0.08 min) (Table 1).

### Optimization of parameters for Full MS/data-dependent MS^2^ (TOP *N*) acquisition

Acquisition of data in Full MS/ddMS^2^ TOP *N* mode allows in addition to the determination of the exact mass of the detected lipid species by HRMS their characterization based on characteristic product ion spectra. In this mode, one full scan (the survey scan) is recorded, followed by the acquisition of a distinct number (*N*) of product ion spectra of selected precursor ions. For reliable analysis of lipids using data-dependent acquisition, it is important to balance between (i) the acquisition of as many product ion spectra as possible to characterize as many precursor ions as possible and (ii) an appropriate cycle time (frequency) enabling to record enough data points (12 to 20) for the semi-quantification in Full MS mode.

The effect of the number of triggered ddMS^2^ (TOP *N*) on the data points across the chromatographic peaks of different labeled lysophospholipids and phospholipids eluting over the chromatographic range was investigated. As expected, with increasing number of the TOP *N*, less data points in Full MS were recorded, and TOP 20 resulted in an insufficiently low number of data points (i.e., 3 to 11 points) per peak (Table 2). For analytes with narrow peaks, e.g., the early eluting lysophospholipids with a FWHM of 3 s, only the TOP 5 acquisition led to sufficient data points (i.e., 13 points). However, for later eluting phospholipids, this resulted in a high number of data points (> 31 points) due to their broader peaks (FWHM >8 s, Table 2). Thus, a higher TOP *N* was chosen for phospholipids yielding more comprehensive qualitative data without forfeiting peak accuracy for quantification. Consequently, the number of ddMS^2^ triggered was adjusted in relation to the FWHM determined for the IS taking the cycle time into account using for Full MS *R* = 60,000 and for ddMS^2^ *R* = 15,000, i.e., 0.8 s for TOP 5 (1 Full MS + 5 ddMS^2^), 1.4 s for TOP 10 (1 Full MS + 10 ddMS^2^), and 2.2 s for TOP 15 (1 Full MS + 15 ddMS^2^). For the elution window of the lysophospholipids (i.e., 0–7 min) TOP 5 was selected; TOP 10 precursor ions were triggered from 7 to 12 min covering phospholipids eluting with a FWHM of 8–10 s; and from 12 min until the end of the analysis TOP 15 was chosen covering phospholipids eluting with a FWHM > 11 s. As this study focuses on phospholipids, no optimization was done for the late-eluting neutral lipids (i.e., DG, Chol Ester, TG). Because one MS^2^ spectrum per lipid species, ideally at the apex of the peak, is sufficient for characterization, the apex trigger was set from 1 to 4 s and the dynamic exclusion to 4 s allowing on the one hand the acquisition of meaningful spectra at high intensity of the precursor ions and on the other hand the trigger of many different precursor ions. So far, the number of MS^2^ scans (TOP *N*) triggered during one duty cycle was not split into segments over the analysis time in previous studies. Instead, they used constant TOP *N* ranging from 2, 3, 10, or 20 for the whole analysis time [7–11, 34, 35, 39, 41, 42]. Our data shows that the optimization of this parameter is crucial to acquire as many product ion spectra as possible for characterization while keeping sufficient data points for semi-quantification in Full MS. The use of an exclusion list covering common contaminants, such as polysiloxanes, alkane polymers, or phthalates, prevents the acquisition of ddMS^2^ of abundant background ions containing no useful information (Table S1). Additionally, using an inclusion list comprising lipids of interest, i.e., PC and PE bearing biologically relevant PUFA (Table S2), ascertains that their ddMS^2^ are recorded and thus reliably allows the characterization of their presence in biological samples.

**Table 2:** Data points across the peaks using Full MS/data-dependent MS^2^ (TOP *N*) acquisition. Shown are the numbers of MS scans across the full chromatographic peak width using different numbers of data-dependent triggered fragment spectra (TOP *N*), and the full width at half maximum (FWHM) (mean ± SD, *n* = 4) for selected deuterium-labeled IS. Resolution settings were Full MS: *R* = 60,000; ddMS^2^: *R* = 15,000. Bold values indicate the TOP *N* selected for the optimized method

	ESI(-)				
Analyte	MS scans over the full peak width				FWHM ± SD [s]
	Top 5	Top 10	Top 15	Top 20	
LPC 18:1[D7]/0:0	**13**	7	4	3	2.93 ± 0.13
LPE 18:1[D7]/0:0	**13**	7	5	3	3.17 ± 0.07
PI 15:0/18:1[D7]	32	**17**	12	9	10.6 ± 0.53
PS 15:0/18:1[D7]	34	**17**	11	7	10.5 ± 0.41
PG 15:0/18:1[D7]	34	**15**	11	8	8.03 ± 0.86
SM 18:1;2O/18:1[D9]	44	21	**14**	11	13.2 ± 0.48
PC 15:0/18:1[D7]	44	23	**15**	11	13.4 ± 1.00
PE 15:0/18:1[D7]	41	21	**13**	9	11.7 ± 0.60

### Optimization of normalized collision energy

In order to obtain meaningful product ion spectra enabling comprehensive characterization of the lipid class as well as the fatty acyl chains, we investigated the influence of the collision energy (CE) on MS^2^ spectra in both ionization modes (Fig. S2). Lipids present in biological samples cover a wide range of masses. Thus, the NCE was used for fragmentation which represents the CE relative to *m/z* 500 and applies an adjusted actual CE depending on the *m/z* of the precursor ion, instead of applying the identical absolute CE regardless of the *m/z*. MS^2^ spectra of the selected PC and PE standards were recorded in both ionization modes using a NCE of 10, 20, 25, or 30, and evaluated for characteristic fragments comprising in ESI(+) the fragments of the phospholipids’ polar head group or its (partly) loss, and in ESI(-) the fragments resulting from the fatty acyl chains [30].

With a NCE of 10, only slight fragmentation was observed: MS^2^ spectra were dominated by the molecular ions and only fragments of the polar head group were observed for both phospholipids in both ionization modes (Fig. S2 A-B, top). With a NCE of 20 and 25, the intensity of the fragments related to the polar head group increased (i.e., *m/z* 184.0733 for PC, and [M+H-141.0191]^+^ for PE) in ESI(+). Moreover, in ESI(-), fragments of the fatty acyl chains were detected (Fig. S2 A-B, middle). Further increasing the NCE to 30 decreased the absolute intensity of most characteristic fragments particularly of those with a high *m/z*.

Based on these findings, a stepwise fragmentation of the precursor ions using a combination of NCE 20 and 25 for both ionization modes was applied in the final method. This is in line with previous studies using also a stepwise fragmentation of 20 and 25 in both ionization modes [39], or 20 and 25 in ESI(+) and 20, 24, and 28 in ESI(-) [8].

### Sample preparation

Selection of suitable extraction conditions for lipids from biological samples is a key prerequisite in untargeted analysis to ensure coverage of lipids with varying polarity. In the present study, individual lipid species were semi-quantified using one IS per lipid class.

Recoveries between the MeOH/MTBE [23] and the two-stepped extraction protocol [25] were comparable for all phospholipid classes covered by the IS except for PS (Table 3). Overall, apparent extraction recovery was good being slightly better in ESI(-) with > 75% except for SM, while in ESI(+) it was > 70% except for LPC and PG. Also, reproducibility of the lipid extraction was excellent with an intra-day and inter-day variance < 100 ± 15% except for PG and SM (< 100 ± 20%). Extraction recovery of PS was considerably lower with the two-stepped extraction (< 13% two steps *vs.* > 80% MeOH/MTBE) and showed high variation, i.e., intra-day variance >100 ± 47% and inter-day variance > 100 ± 97%, in both ionization modes. This poor recovery of PS with the two-stepped extraction is likely due to the addition of acetic acid leading to the protonation of the serine head group and thus the PS (partially) remains in the aqueous phase. With both processing methods, also the neutral lipids, i.e., DG, TG, and Chol Ester, are extracted. However, if also these more hydrophobic lipids are in the focus of analysis, a less polar reconstitution solvent after LLE must be chosen to ensure a better solubilization [32].

**Table 3:** Extraction recovery of IS from human plasma. IS were spiked to three different pools of human plasma prior to extraction and samples were extracted by LLE on three different days using a one-step MeOH/MTBE extraction or a two-stepped extraction with IPA/*n*-hexane//CHCl_3_/MeOH. Shown are the mean values and relative standard deviation (RSD) (*n* = 9 for intra-day, *n* = 27 for inter-day)

ESI(-)	Day 1				Day 2				Day 3				Days 1–3	
	MeOH/MTBE		IPA/*n*-hexane//CHCl_3_/MeOH		MeOH/MTBE		IPA/*n*-hexane//CHCl_3_/MeOH		MeOH/MTBE		IPA/*n*-hexane//CHCl_3_/MeOH		MeOH/MTBE	IPA/*n*-hexane//CHCl_3_/MeOH
Analyte	Recovery [%]	RSD [%]	Recovery [%]	RSD [%]	Recovery [%]	RSD [%]	Recovery [%]	RSD [%]	Recovery [%]	RSD [%]	Recovery [%]	RSD [%]	RSD_interday_ [%]	RSD_interday_ [%]
LPC 18:1[D7]/0:0	87	3	85	9	82	6	75	8	80	4	76	6	6	10
LPE 18:1[D7]/0:0	90	4	73	8	86	6	78	5	86	5	75	7	5	7
PI 15:0/18:1[D7]	108	5	115	8	105	6	120	5	105	4	108	9	5	9
PS 15:0/18:1[D7]	93	5	4	50	89	9	12	90	91	10	22	57	8	97
PG 15:0/18:1[D7]	88	6	104	8	84	11	87	6	88	19	96	7	13	10
SM 18:1;2O/18:1[D9]	69	9	74	8	70	7	63	10	72	7	68	7	8	11
PC 15:0/18:1[D7]	79	3	93	6	78	5	85	5	82	5	84	7	5	7
PE 15:0/18:1[D7]	82	3	91	3	81	5	94	5	85	5	91	4	5	4
ESI(+)	Day 1				Day 2				Day 3				Days 1–3	
	MeOH/MTBE		IPA/*n*-hexane//CHCl_3_/MeOH		MeOH/MTBE		IPA/*n*-hexane//CHCl_3_/MeOH		MeOH/MTBE		IPA/*n*-hexane//CHCl_3_/MeOH		MeOH/MTBE	IPA/*n*-hexane//CHCl_3_/MeOH
Analyte	Recovery [%]	RSD [%]	Recovery [%]	RSD [%]	Recovery [%]	RSD [%]	Recovery [%]	RSD [%]	Recovery [%]	RSD [%]	Recovery [%]	RSD [%]	RSD_interday_ [%]	RSD_interday_ [%]
LPC 18:1[D7]/0:0	69	5	72	14	67	5	63	6	57	5	55	7	10	15
LPE 18:1[D7]/0:0	89	7	73	7	80	5	71	8	80	4	68	6	7	8
PI 15:0/18:1[D7]	101	7	81	9	96	4	80	6	91	6	80	12	7	9
PS 15:0/18:1[D7]	85	10	3	47	81	13	8	92	78	9	21	61	11	106
PG 15:0/18:1[D7]	65	15	75	11	57	18	76	8	61	15	58	9	16	15
SM 18:1;2O/18:1[D9]	122	7	107	11	115	4	107	6	78	8	84	5	20	14
PC 15:0/18:1[D7]	85	5	94	9	79	3	84	6	73	4	75	4	8	11
PE 15:0/18:1[D7]	126	4	117	9	115	7	129	12	107	9	112	8	10	12

**Fig. 3:**
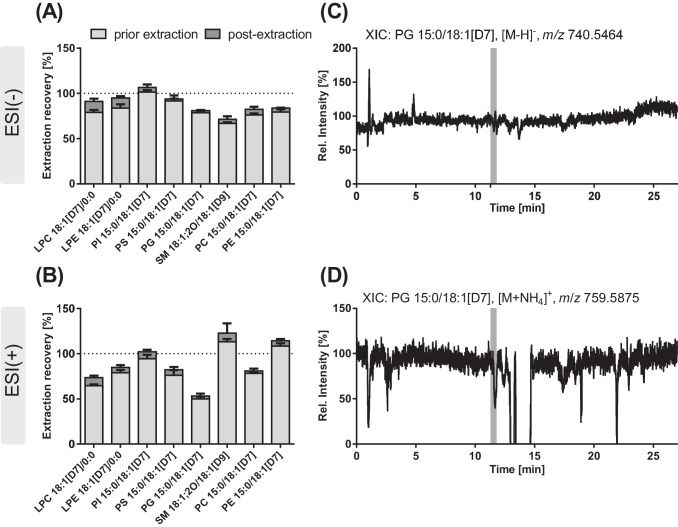
Influence of ion suppression on the extraction recovery from plasma. Shown are the recoveries of deuterium-labeled IS from the extraction of 10 µL of human plasma **A** in ESI(-) and **B** in ESI(+) mode. IS were spiked to the plasma at the beginning of sample preparation (prior extraction) or after sample preparation directly before measurement (post extraction). Shown are mean values ± SD, *n* = 3. **C**, **D** An IS solution (1.9 µM, 5 µL/min) was post-columnly mixed with the LC flow (260 µL/min) following injection of a plasma extract. Shown are the XIC of PG 15:0/18:1[D7] in **C** ESI(-) and** D** ESI(+) mode. The gray bar indicates the retention time of PG 15:0/18:1[D7]

All in all, the MeOH/MTBE-based LLE is more environmentally friendly (no halogenated solvents) and the collection of the upper phase containing the lipids is easier in comparison to the extraction with CHCl_3_ where the lipids are in the lower phase. Moreover, it showed better extraction of the PS lipid class. In consequence, the MeOH/MTBE extraction was selected and further characterized. Regarding robustness, the extraction recoveries were not impacted by the plasma pool used for LLE (Fig. S3). Inter-operator variability was thus determined combining all data from three different days and plasma pools (*n* = 81), and was excellent (< 100 ± 12% in ESI(-) and < 100 ± 23% in ESI(+)). Only the extraction of PS was affected by the operator, showing a recovery < 56% when samples were prepared by operator 2, while > 80% were recovered by operators 1 and 3, which is reflected by an inter-operator variance of 100 ± 33%. Comparison of extraction recoveries of IS spiked to plasma prior to or post extraction unveiled that apparent losses of IS during sample preparation are < 15% for the lysophospholipids and < 8% for the phospholipids in both ionization modes (Fig. 3A, B). These losses during sample preparation were slightly better than those reported for a modified Matyash protocol with an average loss of 27% [43]. Low apparent recoveries were observed particularly for PG 15:0/18:1[D7] in ESI(+) (50%) when the IS was added after extraction. These losses in the signal thus occur during LC–MS analysis due to ion suppression.

### Ion suppression analysis

Ion suppression analysis showed in ESI(+) a strong suppression of the signal of PG 15:0/18:1[D7] (approx. 50%) at its elution time, while in ESI(-) the signal was less affected. This confirms the results of the spiking experiments and might explain the higher variance of the recovery of PG 15:0/18:1[D7] (Fig. 3, Table 3, Fig. S3). Overall, ion suppression analysis revealed stronger ion suppression effects in ESI(+) compared to ESI(-), and was similar for the different investigated plasma pools. Besides ion suppression, also ion enhancement effects were observed at the corresponding elution times, e.g., for SM 18:1;2O/18:1[D9] and PE 15:0/18:1[D7] in ESI(+), and for PI 15:0/18:1[D7] in ESI(-) (not shown).

Extraction of a higher plasma volume (i.e., 20 and 50 µL) increased the ion suppression effects in ESI(+), especially for SM 18:1;O2/18:1[D9]. In ESI(-), besides ion suppression, strong ion enhancement was observed for PI 15:0/18:1[D7] and PG 15:0/18:1[D7] with higher plasma volume (Fig. S4). Thus, the use of 10 µL plasma is preferred as here ion suppression effects of the IS were acceptable. This sample volume is in line with previous lipidomics methods, e.g., Wang *et al.* and Chen *et al.* used 10 µL of human plasma for the extraction of lipids with a MeOH/MTBE-based LLE [44, 45] and Ottestad *et al.* extracted lipids from 10 µL of human plasma using a mixture of CHCl_3_/MeOH [46].

#### Excursus: Deletion of MS signals by orbitrap MS

**Fig. 4:**
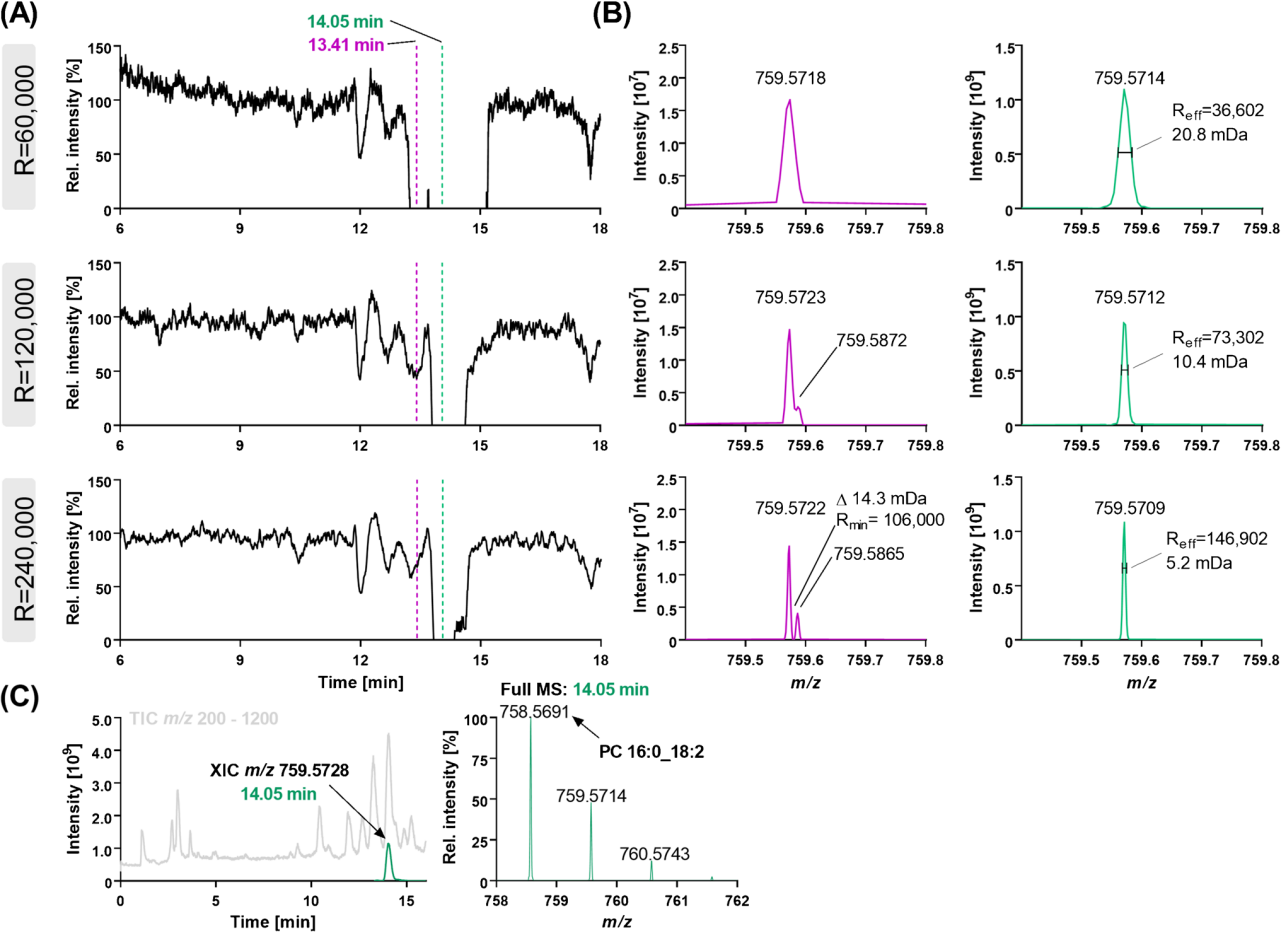
Collapse of the signal at *m/z* 759.5875 (PG 15:0/18:1[D7], [M + NH_4_]^+^) in LC-ESI(+)-HRMS ion suppression analysis. An IS solution was post-columnly mixed with the LC flow following injection of a plasma lipid extract and Full MS spectra were recorded. **A** XIC at *m/z* 759.5875 (PG 15:0/18:1[D7], [M + NH_4_]^+^) using resolution settings of 60,000, 120,000, or 240,000. **B** Full MS spectra at 13.41 min (purple) and 14.05 min (green) showing the resolution-dependent interference of the signal*.* **C** LC–MS chromatogram: TIC (*m/z* 200–1200) and XIC at *m/z* 759.5728 showing the peak of the interference and its Full MS spectrum

Ion suppression analysis unveiled in ESI(+) a complete drop of the signal of PG 15:0/18:1[D7] ([M + NH_4_]^+^ *m*/*z* 759.5875) to zero on two retention times between 13.24 and 15.17 min (for 116 s) (Fig. 3D and Fig. 4). Increasing the resolution setting of the Full MS scan from 60,000 to 240,000 decreased this signal drop to 32 s, but it still occurred even at the highest resolution (Fig. 4A). Full MS spectra indicate a distortion of the *m/z* by an interfering analyte resulting in a mass deviation > 20 ppm for the *m/z* of PG 15:0/18:1[D7] ([M + NH_4_]^+^) at both retention times (Fig. 4B). Based on the *m/z* determined in the lipid extract without IS infusion, as well as its MS^2^ spectra, the interference could be related to the [^13^C_1_] isotope of PC 16:0_18:2 ([M + H]^+^ *m/z* 759.5728) which is present at high abundance in the plasma lipid extract (Fig. 4C). The elution window of this analyte from 13.30 to 15.01 min fits to the deletion of the signal. The separation of this interfering *m/z* was achieved at 13.41 min with a resolution of 240,000, but this was not the case around the chromatographic apex of the peak (i.e., 14.05 min) (Fig. 4B, bottom).

Using a standard solution of PC 16:0/18:2(9Z,12Z) instead of a lipid extract for the ion suppression analysis confirmed that the distortion of the signal of PG 15:0/18:1[D7] was caused by PC 16:0/18:2(9Z,12Z) ions at high abundance: When 6 µM of the PC 16:0/18:2(9Z,12Z) was injected, the signal of PG 15:0/18:1[D7] at 14.05 min was distorted, while decreasing the injected concentration of the PC 16:0/18:2(9Z,12Z) to a similar intensity of the ions at *m/z* 759.5728 and *m/z* 759.5875 allowed the parallel detection of both *m/z* (Fig. S5). The distortion of the *m/z* also depended on the amount of ions and the filling of the orbitrap (defined by the AGC target): When the ratio of PC 16:0/18:2(9Z,12Z) : PG 15:0/18:1[D7] was kept constant (12 : 1), a high concentration (3–6 µM) and a high number of ions in the trap (AGC ≥ 1 × 10^6^) led again to the distortion of the signal of PG 15:0/18:1[D7]. At a lower filling of the trap (AGC target 2 × 10^4^) both *m/z* were detected. In contrast, at low concentration (0.6 µM) only with a higher AGC target (≥ 1 × 10^6^) both *m/z* were detected (Fig. S6). Similar findings of distortion of the *m/z* signal by abundant almost isobaric ions (despite sufficient resolution) have already been reported in orbitrap MS and can be explained by the formation of ion clouds with almost identical *m/z* within the trap resulting in peak coalescence [47]. Overall, this shows that when using an orbitrap mass analyzer abundant lipid species influence the detection of lower abundant ones with similar *m/z* which should be considered during method development, i.e., (i) choosing the highest resolution possible for Full MS analysis, and (ii) using an efficient chromatographic separation — as described herein — to separate almost isobaric lipids.

Overall, the LLE with MeOH/MTBE yielded excellent extraction recoveries of the lysophospholipids and phospholipids, and apparent losses resulted mainly from ion suppression. 10 µL of plasma was chosen for LLE to limit matrix effects and to enable semi-quantification. Intra- and inter-day variance < 100 ± 20%, as well as inter-operator variance < 100 ± 18% (except for PS and PG) using different pools of plasma demonstrate the reproducibility and robustness of the extraction method underlining its suitability for lipidomics analysis (Fig. S3). The observed signal erase for PG due to the [^13^C_1_] of PC 16:0_18:2 in ESI(+) does not impact the detection of PG 15:0/18:1[D7] because it elutes earlier at 11.43 min (Table 1). However, it should be kept in mind that the detection of lipid species with nearly identical *m/z* eluting at the same time might be disturbed using FT-MS instruments resulting in loss or distortion of information regarding their presence. This is particularly an issue in shotgun analysis where all ions enter the MS at the same time, but also might occur to some extent when combined with chromatographic separation. The use of a higher resolution might reduce this effect, but cannot prevent deletion of the signal of minor lipid species by dominating ions with similar *m/z.*

### Application: Analysis of the effects of n3-PUFA supplementation on the human plasma lipidome

**Fig. 5:**
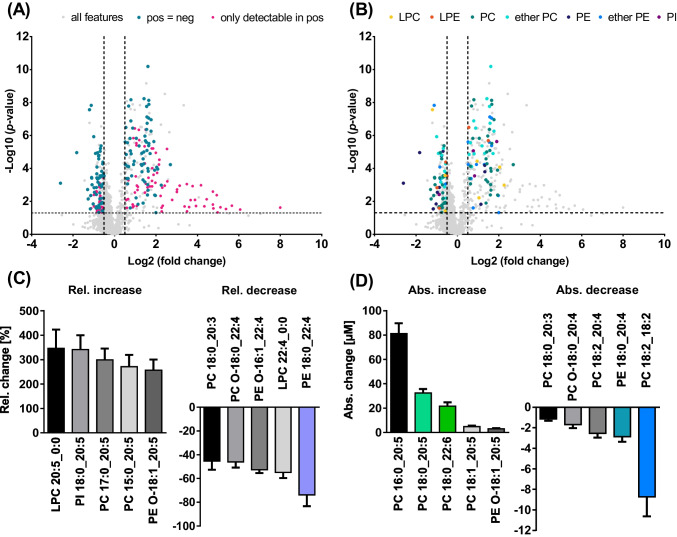
Untargeted LC-HRMS analysis of phospholipids in human plasma following n3-PUFA supplementation. Plasma lipid extracts at baseline and following 12 months of n3-PUFA supplementation were analyzed by untargeted LC-HRMS. **A** Shown are volcano plots highlighting significantly changing features detected in both ionization modes and those only detectable in ESI(+). **B** Significantly changing features detected in both modes are highlighted according to their lipid class. Shown are the **C** relative and **D** absolute changes observed in ESI(+) for the TOP 5 most changing tentatively identified lipids (mean ± SEM, *n* = 20). Highlighted lipids were further quantified by targeted LC–MS/MS

The effects of 12-month n3-PUFA supplementation (corresponding to 4 portions of fatty fish per week) on the plasma phospholipid pattern in healthy subjects were investigated aiming to characterize which phospholipids are changing most following supplementation. After manual review of the feature assignment by MS-DIAL, 1399 features in ESI(+) and 580 in ESI(-) were found and evaluated by means of volcano plots (Fig. 5A). For evaluation of which lipid species showed a relevant change after n3-PUFA supplementation, only features with a − log10 (*p*-value) ≥ 1.303 (corresponding to − log10 (*p* ≤ 0.05)) and a log2 (fold change) ≥ 0.5 or ≤  − 0.5 were further investigated (348 features in ESI(+) and 151 in ESI(-)).

From these, only the features detected in both ionization modes were semi-quantified aiming to (i) characterize the lipid class and the fatty acyl chains of the lipids, which was not done in previous lipidomics studies investigating n3-PUFA supplementation [45, 46, 48], and to (ii) provide additional confidence regarding the tentative identification of the lipid species and their modulation following n3-PUFA supplementation by comparing the results from both ionization modes. Thus, DG, TG, and Chol Ester were not further evaluated (Fig. 5A). Further confidence of the lipid identification was obtained utilizing retention time behavior of lipids in RPLC which correlates with the equivalent carbon number. Plotting the retention times of the logical series of lipid species *vs.* the carbon number of the fatty acyl chains (Fig. S9) or *vs.* the number of double bonds (Fig. S10) showed good correlation of polynomial regression for the different lipid classes [12]. A total of 98 lipid species were semi-quantified based on their peak height using the IS of the same lipid class and concentrations obtained from the analysis in ESI(+) and ESI(-) mode were comparable (Table S5). For the TOP 5 most changing lipids (relatively and absolutely) (Fig. 5C, D), additionally, the concentrations for each subject are given in the supplementary information (Table S6).

Significant changes following n3-PUFA supplementation were observed for PC, ether PC, ether PE, PE, LPE, PI, and LPC lipid species. With 34 lipids, most changes were observed for the PC lipid class followed by ether PC, ether PE, and PE with 24, 15, and 11 species, respectively (Fig. 5B). Following n3-PUFA supplementation, 8 PE species decreased, while 11 ether PE species increased. The change of a higher number of PC species is likely related to the high abundance of PC in human plasma being the most abundant class of phospholipids accounting for around 18% of all lipids [49, 50]. This is in line with an earlier analysis of the samples from the same study where Browning *et al.* also found the strongest increase of DHA + EPA in the PC fraction among the different analyzed plasma lipid fractions (i.e., PC, Chol Ester, and TG). However, other phospholipid classes were not analyzed [28].

Following n3-PUFA supplementation, the most increasing lipids bear 20:5 (Fig. 5C). It should be noted that the baseline concentration of lipids bearing 20:5 was lower than that of lipids bearing 22:6, which is consistent with a previous study reporting a lower total fatty acid level of 20:5(5Z,8Z,11Z,14Z,17Z) compared to 22:6(4Z,7Z,10Z,13Z,16Z,19Z) (40 ± 28 µM and 89 ± 37 µM, respectively) in plasma from healthy Canadian adults [51]. Due to their lower baseline concentration, a stronger increase was observed for lipids bearing 20:5: For example, LPC 20:5_0:0 was less abundant at baseline than LPC 22:6_0:0 (i.e., 0.25 ± 0.11 µM *vs.* 0.80 ± 0.28 µM) and was relatively increased by 350%, while LPC 22:6_0:0 showed an increase of 100%. Similar results were also observed for other phospholipid classes, i.e., for the pairs PI 18:0_20:5/PI 18:0_22:6, PC 17:0_20:5/PC 17:0_22:6, LPE 20:5_0:0/LPE 22:6_0:0, and PC O-18:1_20:5/PC O-18:1_22:6. These observations are in line with results from the same trial showing that the fatty acid 20:5(5Z,8Z,11Z,14Z,17Z) [52] and its oxylipin products (e.g., 14(15)-EpETE) [53] are relatively more affected by n3-PUFA supplementation compared to 22:6(4Z,7Z,10Z,13Z,16Z,19Z) and its oxylipin products. Our results are consistent with previous lipidomics studies showing that LPC 20:5_0:0 increased more than LPC 22:6_0:0 following n3-PUFA supplementation [45, 46]. Interestingly, also the determined relative changes for LPC 20:5_0:0 (i.e., 4.10 *vs.* 4.35) and LPC 22:6_0:0 (i.e., 1.93 *vs.* 1.89) were similar with the studies from Ottestad *et al.* where healthy subjects (*n* = 16) received fish oil capsules containing 0.7 g EPA + 0.9 g DHA per day for 7 weeks, indicating a steady state of modulation [46].

The strongest relative decrease was observed for lipids bearing 22:4 with, e.g., PE 18:0_22:4 showing a decrease of − 74% and LPC 22:4_0:0 of − 55% (Fig. 5C), and those bearing 20:3 with, e.g., PC 18:0_20:3 decreasing by − 45%. Other lipids bearing potential n6-PUFA, such as ARA (20:4(5Z,8Z,11Z,14Z)) or linoleic acid (18:2(9Z,12Z)), also decreased following n3-PUFA supplementation, e.g., PE 18:0_20:4 with − 37% and PC 18:2_18:2 with − 27%. Interestingly, the latter showed also the strongest decrease in concentration (Fig. 5D). These results are in line with previous studies reporting that n3-PUFA supplementation led to a decrease of the n6-PUFA content [28, 52, 54, 55]. Our results are also consistent with the study from Uhl *et al.* where a significant decrease was found for PC 16:0/22:4, PC 18:0/22:4, and PC 18:1/20:4 in plasma from subjects supplemented with 510 mg/day of DHA for 29 days by means of targeted lipidomics analysis [56].

**Fig. 6:**
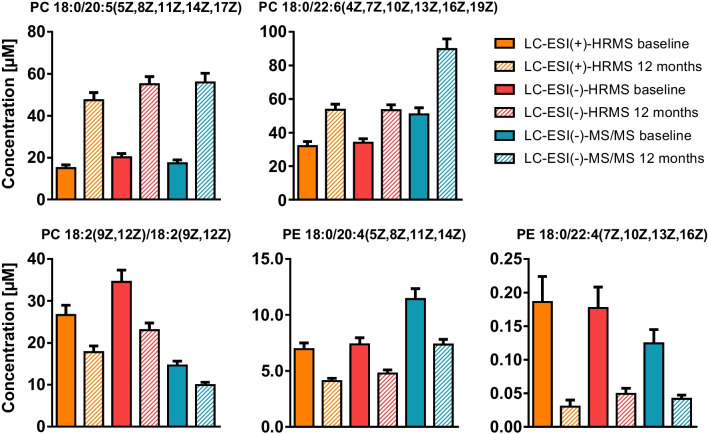
Comparison of concentrations of selected phospholipids determined by means of untargeted LC-HRMS and targeted LC–MS/MS. Plasma lipid extracts were analyzed by untargeted LC-HRMS and lipids were semi-quantified based on peak heights using one IS per lipid class. For quantification, the extracts were analyzed by targeted LC-ESI(-)-MS/MS and lipids were quantified by external calibration based on analyte to corresponding IS peak area ratios. Shown are the concentrations of selected phospholipids at baseline and following 12 months of n3-PUFA supplementation (mean ± SEM, *n* = 20)

In order to support the results determined with the untargeted approach, several of the most changing phospholipids in the plasma were quantified using targeted LC–MS/MS. Targeted analysis confirmed the tentative identification of the phospholipids PC 18:0/20:5(5Z,8Z,11Z,14Z,17Z), PC 18:0/22:6(4Z,7Z,10Z,13Z,16Z,19Z), PE 18:0/20:4(5Z,8Z,11Z,14Z), PE 18:0/22:4(7Z,10Z,13Z,16Z), and PC 18:2(9Z,12Z)/18:2(9Z,12Z) (Table 4, Fig. S11). Concentrations determined by quantitative targeted LC–MS/MS were in the same range as those semi-quantified by means of LC-HRMS, also confirming the observed increase and decrease following n3-PUFA supplementation (Fig. 6). Quantitative targeted analysis resulted in almost same concentration for PC 18:0/20:5(5Z,8Z,11Z,14Z,17Z), while < 2.5-fold difference in concentrations was found for PC 18:0/22:6(4Z,7Z,10Z,13Z,16Z,19Z), PE 18:0/20:4(5Z,8Z,11Z,14Z), PE 18:0/22:4(7Z,10Z,13Z,16Z), and PC 18:2(9Z,12Z)/18:2(9Z,12Z).

**Table 4:**
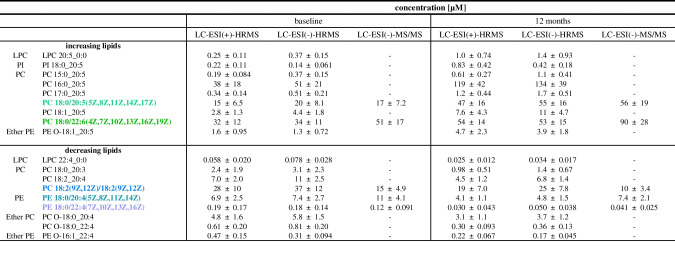
Concentration of selected phospholipids determined by untargeted and targeted analysis in plasma at baseline and following 12 months of n3-PUFA supplementation. Plasma lipid extracts were analyzed by untargeted LC-HRMS in both ionization modes and lipids were semi-quantified based on normalized peak heights using one IS per lipid class. For quantification, extracts were analyzed by targeted LC-ESI(-)-MS/MS and lipids were quantified by external calibration based on analyte to corresponding IS peak area ratios. Shown are mean values ± SD, *n* = 20. The highlighted lipids were among the most changing species determined by means of untargeted LC-HRMS (see Fig. 5C, D)

This supports the suitability of the developed untargeted LC-HRMS method to monitor changes in the phospholipid pattern in plasma. Our results showed a significant increase of lipids bearing n3-PUFA combined with a decrease of lipids bearing n6-PUFA [28, 52, 54]. PC 18:0/20:5(5Z,8Z,11Z,14Z,17Z) and PC 18:2(9Z,12Z)/18:2(9Z,12Z) were found to be significantly modulated and might be further investigated as possible biomarkers for n3-PUFA consumption.

## Conclusion

We optimized and characterized the performance of an LC-HRMS method for the semi-quantification of polar lipids in human plasma. Individual MS parameters were systematically optimized allowing a threefold gain in sensitivity. It was shown that the setting of the auxiliary gas is critical for the spray stability and the S-lens RF level has a relevant impact on the ion transmission and signal intensity. Optimization of chromatographic parameters showed that the selection of the RP C18 column has a great impact on the separation efficiency and peak shape, indicating that surface interactions play a relevant role on phospholipid separation in RPLC.

Data-dependent MS^2^ settings were identified to be crucial and the number of TOP *N* was adjusted based on the FWHM of the chromatographic peaks over the elution time in a staggered manner. This allows the acquisition of as many meaningful MS^2^ spectra as possible for lipid characterization, while recording enough data points across the peaks for semi-quantification. Of note, we found that high abundant lipids can distort the detection of lipids with similar *m/z* in the orbitrap mass analyzer. Thus, the highest resolution possible should be selected for Full MS analysis; however, this does not resolve the problem completely.

Thorough characterization of matrix effects by pre and post extraction spiking as well as ion suppression analysis of plasma unveiled that apparent losses are caused by ion suppression. Thus, concentrations resulting from semi-quantification based on only one IS per lipid class might be affected by coeluting interferences in RPLC. However, determined concentrations in plasma by untargeted LC-HRMS were comparable in ESI(-) and ESI(+), and in the same range as those obtained by targeted LC–MS/MS.

The developed LC-HRMS method was successfully used to investigate the effects of n3-PUFA supplementation on the phospholipid pattern in human plasma. A total of 98 phospholipids, which were significantly changed following supplementation, were semi-quantified in both ionization modes. A strong increase was found for lipids bearing 20:5, while lipids bearing 22:4, and of note PC 18:2(9Z,12Z)/18:2(9Z,12Z) were lowered. Quantitative targeted analysis confirmed the identification of selected phospholipids and their relative change following n3-PUFA supplementation. The concentration difference between the targeted and the untargeted approach was less than 2.5-fold, underlining the reliability of the semi-quantification using the developed untargeted LC-HRMS method.

### Supplementary information

Below is the link to the electronic supplementary material.

Supplementary file1 (PDF 418 KB)

Supplementary file2 (XLSX 87.1 KB)

## Abbreviations

ACN: Acetonitrile
AGC: Automatic gain control
ARA: Arachidonic acid (20:4(5Z,8Z,11Z,14Z))
CE: Collision energy
Chol Ester: Cholesteryl ester
CXP: Collision cell exit potential
dd: Data-dependent
DG: Diacylglycerol
DHA: Docosahexaenoic acid (22:6(4Z,7Z,10Z,13Z,16Z,19Z))
DP: Declustering potential
EDTA: Ethylenediaminetetraacetic acid
EPA: Eicosapentaenoic acid (20:5(5Z,8Z,11Z,14Z,17Z))
ESI: Electrospray ionization
ESI( +): Positive electrospray ionization
ESI(-): Negative electrospray ionization
FC: Free cholesterol
FWHM: Full width at half maximum
HESI: Heated electrospray ionization
HILIC: Hydrophilic interaction liquid chromatography
HPLC: High-pressure liquid chromatography
HRMS: High-resolution mass spectrometry
IPA: *iso*-Propanol
IS: Internal standard
IT: Injection time
LC: Liquid chromatography
LLE: Liquid-liquid extraction
LLOQ: Lower limit of quantification
LOD: Limit of detection
LPC: Lysophosphatidylcholine
LPE: Lysophosphatidylethanolamine
*m/z*: Mass-to-charge ratio
MeOH: Methanol
MG: Monoacylglycerol
MRM: Multiple reaction monitoring
MS: Mass spectrometry
MTBE: *tert*-Butyl methyl ether
NCE: Normalized collision energy
NPLC: Normal-phase liquid chromatography
PA: Phosphatidic acid
PC: Phosphatidylcholine
PE: Phosphatidylethanolamine
PG: Phosphatidylglycerol
PI: Phosphatidylinositol
PS: Phosphatidylserine
PUFA: Polyunsaturated fatty acid
R: Resolution
RF: Radio frequency
RPLC: Reversed-phase liquid chromatography
RSD: Relative standard deviation
*S*/*N*: Signal-to-noise ratio
SM: Sphingomyelin
*sn*: Stereospecific numbering
SPLASH: Single-vial prepared lipidomic analytical standard for human plasma lipids
TG: Triacylglycerol
TIC: Total ion chromatogram
*t*_R_: Retention time
XIC: Extracted ion chromatogram

## Declarations

Blood was collected from healthy human subjects in accordance with the guidelines of the Declaration of Helsinki and approved by the ethics committee of the University of Wuppertal. The n3-PUFA supplementation study was approved by the Suffolk Local Research Ethics Committee.
