# Supplementary material for: LC-ESI-HRMS — lipidomics of phospholipids: Characterization of extraction, chromatography and detection parameters
Source: Anal Bioanal Chem. 2024 Jan 12;416(4):925–44. doi: 10.1007/s00216-023-05080-0 (PMC10800306; doi:10.1007/s00216-023-05080-0)
Supplement: Supplementary file 1 — Supplementary file1 (PDF 418 KB) [file 216_2023_5080_MOESM1_ESM.pdf]

LC-ESI-HRMS - lipidomics of phospholipids

–

Characterization of extraction, chromatography and detection parameters

Supplementary Information

Katharina M. Rund[1, #], Laura Carpanedo[1, #], Robin Lauterbach[1], Tim Wermund[1], Annette L. West[2], Luca M. Wende[1], Philip C. Calder[2,3], Nils Helge Schebb[1*]

[1] Chair of Food Chemistry, Faculty of Mathematics and Natural Sciences, University of Wuppertal, Wuppertal, Germany

[2] School of Human Development and Health, Faculty of Medicine, University of Southampton, Southampton, United Kingdom

[3] National Institute for Health Research (NIHR) Southampton Biomedical Research Centre, University Hospital Southampton NHS Foundation Trust and University of Southampton, Southampton, United Kingdom

[#] Both authors contributed equally

[*]Contact information of the corresponding author:

Nils Helge Schebb

Chair of Food Chemistry

Faculty of Mathematics and Natural Sciences

University of Wuppertal

Gaussstr. 20

42119 Wuppertal

nils@schebb-web.de

Tel: +49-202-439-3457

Results and discussion – Supplementary Information

Optimization of mass spectrometric parameters

ESI-spray voltage and sheath gas showed only little effect on the signal intensity around the optimal range (Fig. S1). Increasing the spray voltage from |2| to |4| kV increased the signal, but higher values led to a less stable spray. In the optimal range (|3.3|-|3.8| kV), the applied voltage showed only marginal effects on the signal intensity of PE 18:1(9Z)/18:1(9Z) in both ionization modes (≤ 12%), while for PC 16:0/20:4(5Z,9Z,11Z,14Z) the signal intensity was increased by 33% in ESI(+) and 17% in ESI(-). The selected optimized values, i.e., 3.4 and -3.7 kV, are lower compared to the spray voltage used in the application note (i.e., |4.2| kV) and higher compared to the default setting in negative mode (i.e., -2.5 kV). However, our results are in line with previous methods using a Q Exactive HF for the analysis of lipids in rat plasma and liver (1), and in pituitary adenoma tissues (2) with a spray voltage between |3| and |4| kV. Variation of the sheath gas flow (range 10-50 psi) showed for both lipids the same change in signal intensity, having a greater effect in ESI(+). 32 and 28 psi were chosen in ESI(-) and ESI(+), respectively. Using the same flow rate (i.e., 260 µL/min), Hu *et al.* used a higher sheath gas flow of 45 psi in both ionization modes for lipidomics analysis which is similar to the default settings. However, they did not show if and how this value was optimized (2).

The sweep gas flows towards the entrance of the heated ion transfer tube at the sweep cone thus acting as a barrier for non-charged molecules and non-volatiles preventing contamination of the source. Increasing the sweep gas flow (i.e., from 2 to 8 arb) led to a slight increase in signal intensity. However, this resulted also in elevated noise of the signal. The selected sweep gas flow of 2 arb was consistent with previously described methods using a sweep gas flow of 1 arb for lipidomics analysis (1, 3, 4). No noticeable effect on the signal could be observed when varying the capillary temperature (i.e., 200 °C - 350 °C). Thus 250 °C was selected, which is consistent with the range (230 °C – 350 °C) used in other lipidomics methods (1, 3-7).

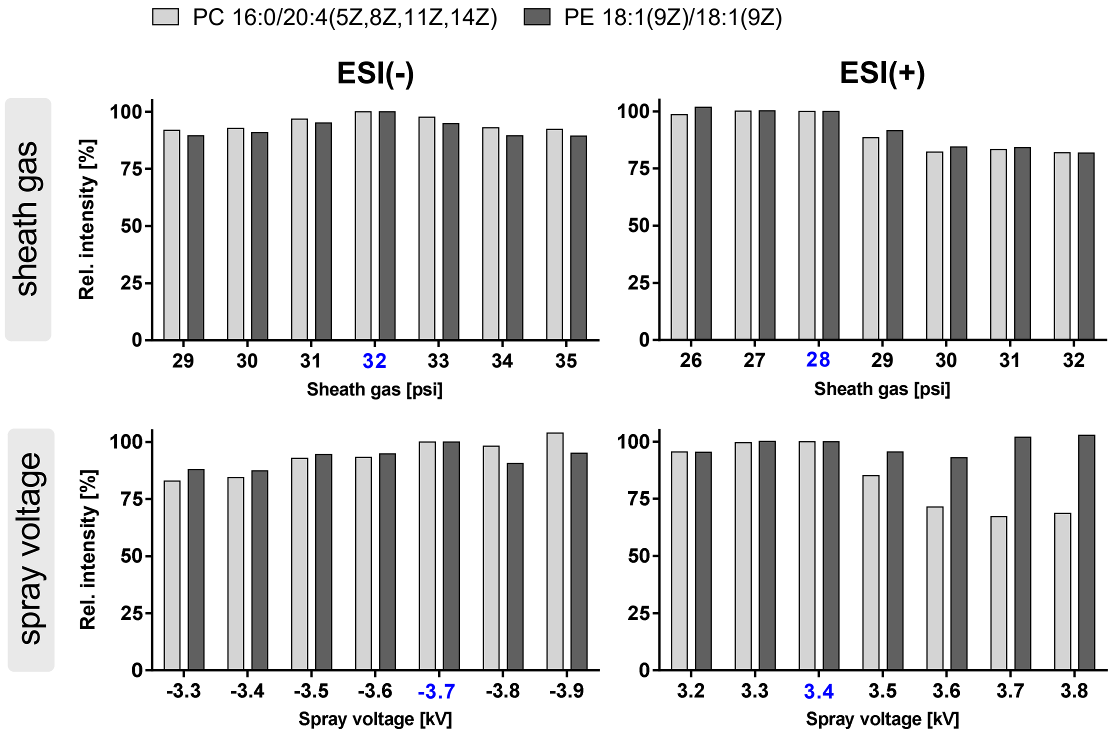

Fig. S1: Effect of selected source parameters on the intensity of the ESI-MS signal. Shown is the influence of **(top)** the sheath gas and **(bottom)** spray voltage on the signal intensity in ESI(-) and in ESI(+) mode for PC 16:0/20:4(5Z,8Z,11Z,14Z) and PE 18:1(9Z)/18:1(9Z) while other parameters were set to default (Fig. 1). Intensities are depicted relative to the signal intensity at the optimized values highlighted in blue.

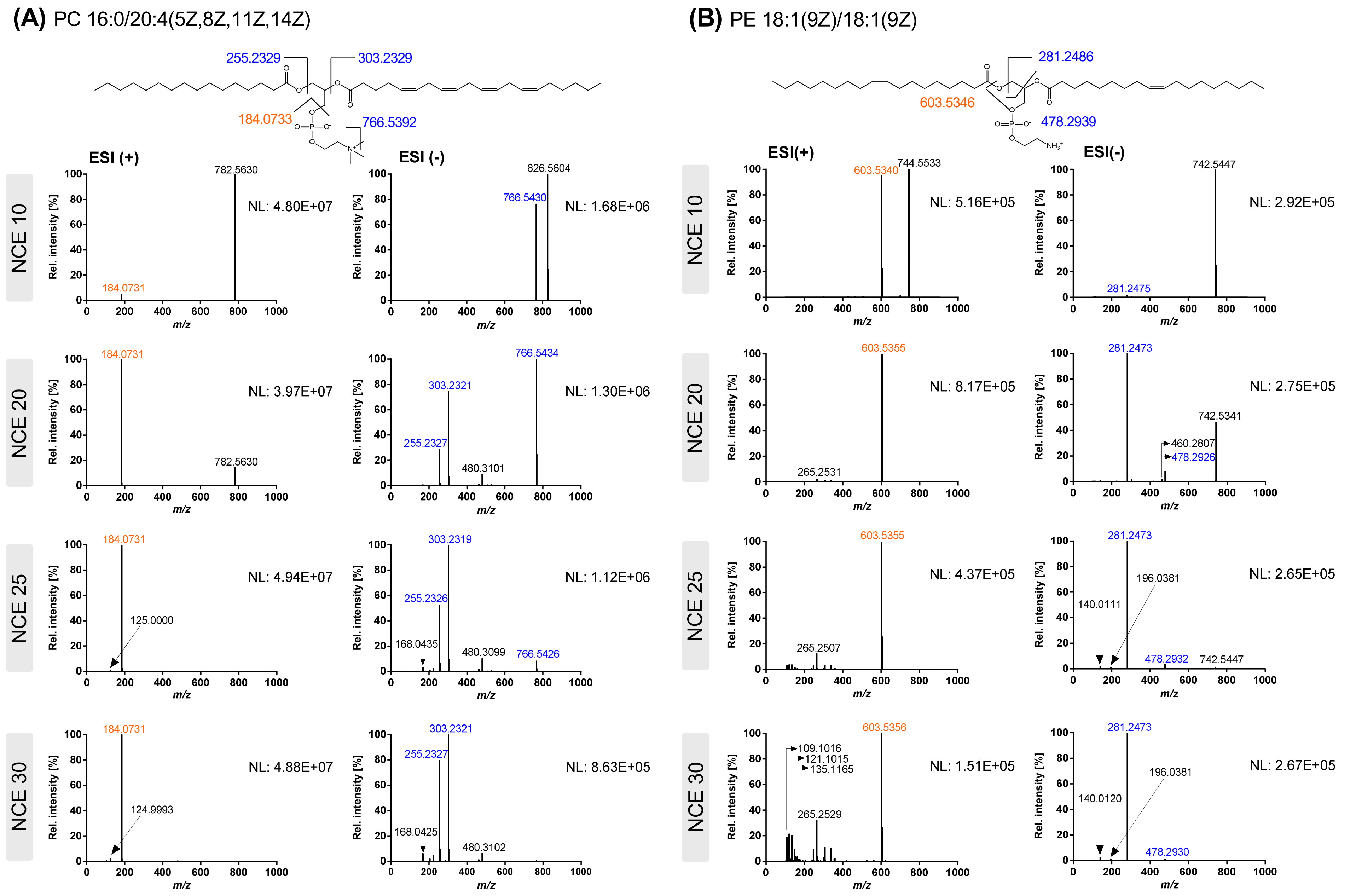

Fig. S2: Product ion spectra of **(A)** PC 16:0/20:4(5Z,8Z,11Z,14Z) and **(B)** PE 18:1(9Z)/18:1(9Z) with different NCE in ESI(+) and ESI(-) mode. The precursor ions were for PC 16:0/20:4(5Z,8Z,11Z,14Z) *m/z* 782.5694 $[M+H]^+$ in ESI(+) and *m/z* 826.5604 $[M+COOH]^-$ in ESI(-) mode, and for PE 18:1(9Z)/18:1(9Z) *m/z* 744.5538 $[M+H]^+$ in ESI(+) and *m/z* 742.5392 $[M-H]^-$ in ESI(-) mode. Suggested sites of fragmentation are indicated in the structures. NL: normalized intensity level, i.e., intensity at 100%.

(A)

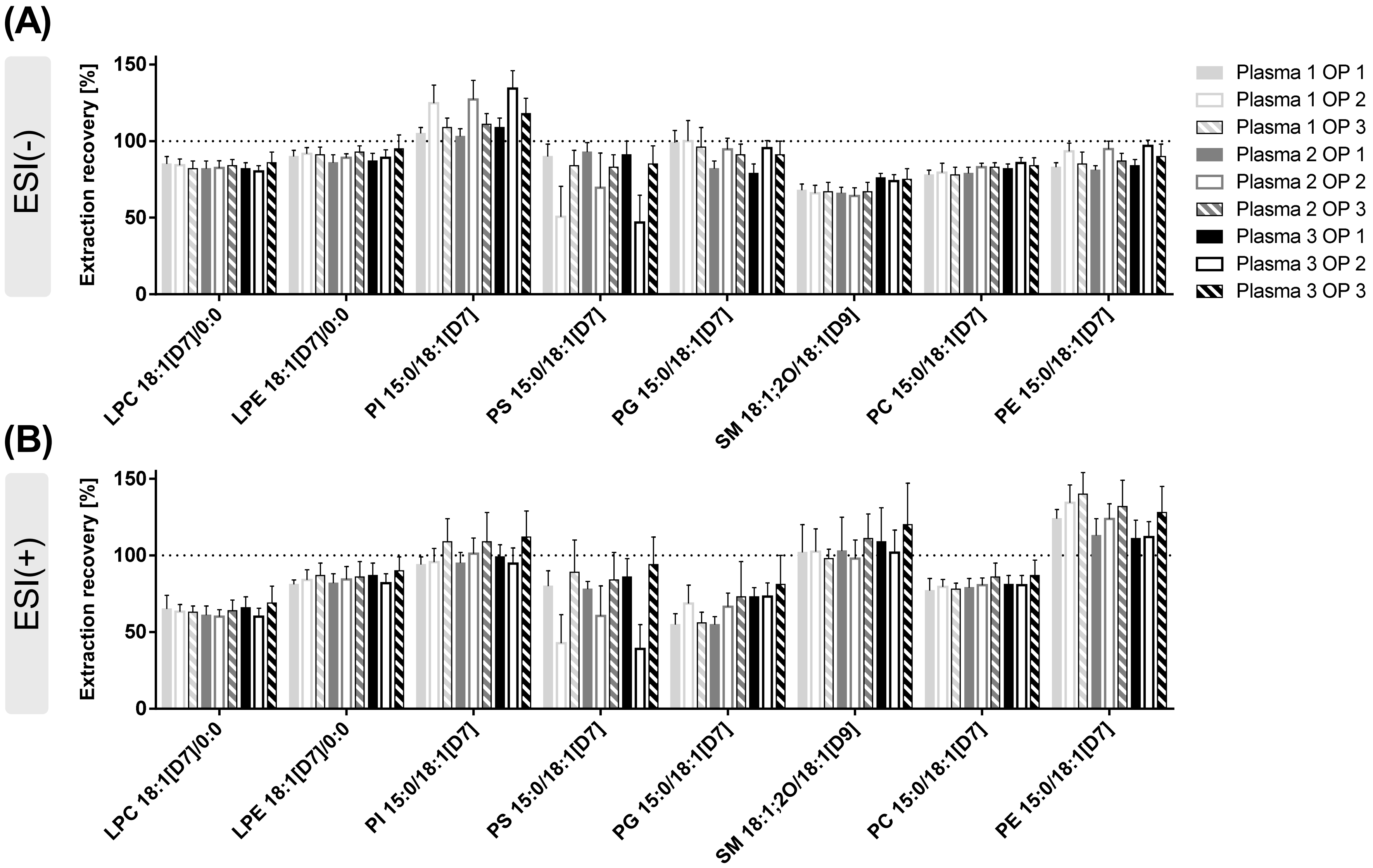

Fig. S3: Robustness of extraction recovery. Shown is the extraction recovery of deuterium-labeled IS from three different pools of human plasma by three different operators on three different days **(A)** in ESI(-) and **(B)** in ESI(+) mode. Shown are mean values ± SD (n=9).

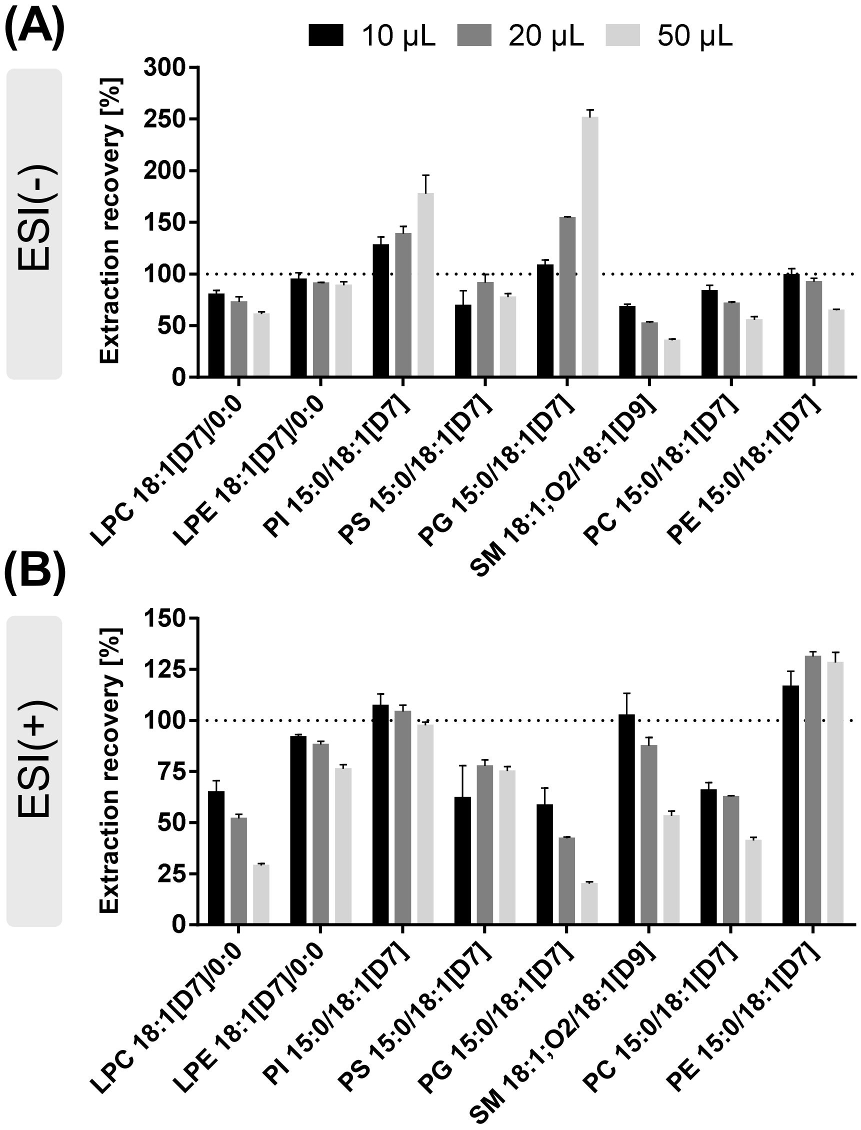

Fig. S4: Influence of the plasma volume on the extraction recovery. Shown is the extraction recovery of deuterium-labeled IS from the extraction of different volumes of human plasma **(A)** in ESI(-) and **(B)** in ESI(+) mode. Extraction recovery was calculated relative to an IS solution directly injected. Shown are mean values ± SD (n=3).

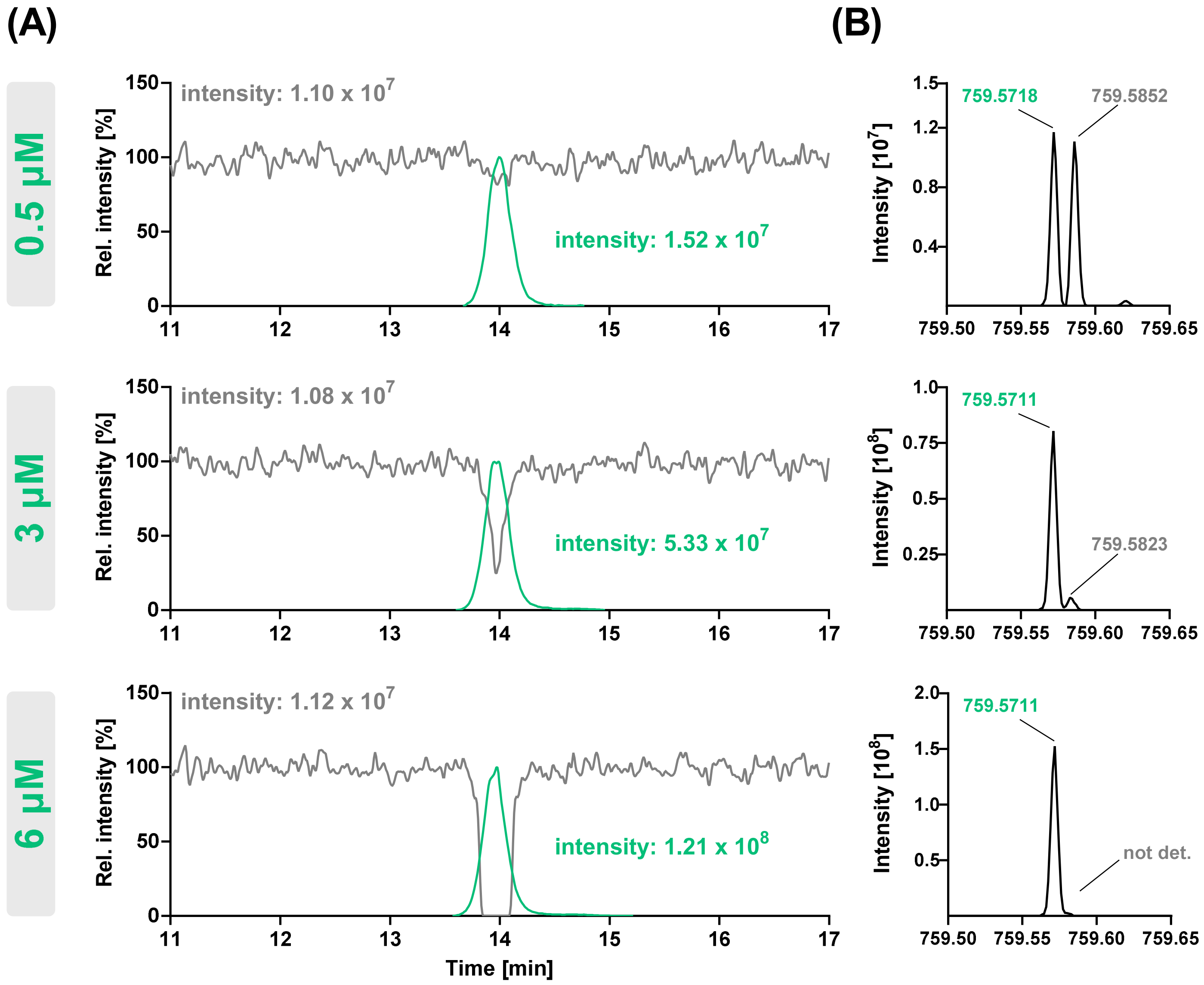

Fig. S5: Ion suppression analysis of PG 15:0/18:1[D7] in ESI(+). A PG 15:0/18:1[D7] solution (1.9 µM, 5 µL/min) was post-columnly mixed with the LC flow following injection (5 µL) of a standard solution of PC 16:0/18:2(9Z,12Z) (0.5 µM (**top**), 3 µM (**middle**) and 6 µM (**bottom**)). (**A**) XIC signal at *m/z* 759.5875 (PG 15:0/18:1[D7], $[M+NH_4]^+$) in gray and in green the peak of ($[^{13}C_1]$ PC 16:0/18:2(9Z,12Z) $[M+H]^+$) at *m/z* 759.5728. **(B)** Full MS spectra at 14.05 min showing the concentration dependent interference of the peak of PG 15:0/18:1[D7] $[M+NH_4]^+$ with the peak of $[^{13}C_1]$ PC 16:0/18:2(9Z,12Z) $[M+H]^+$ with similar *m/z*.

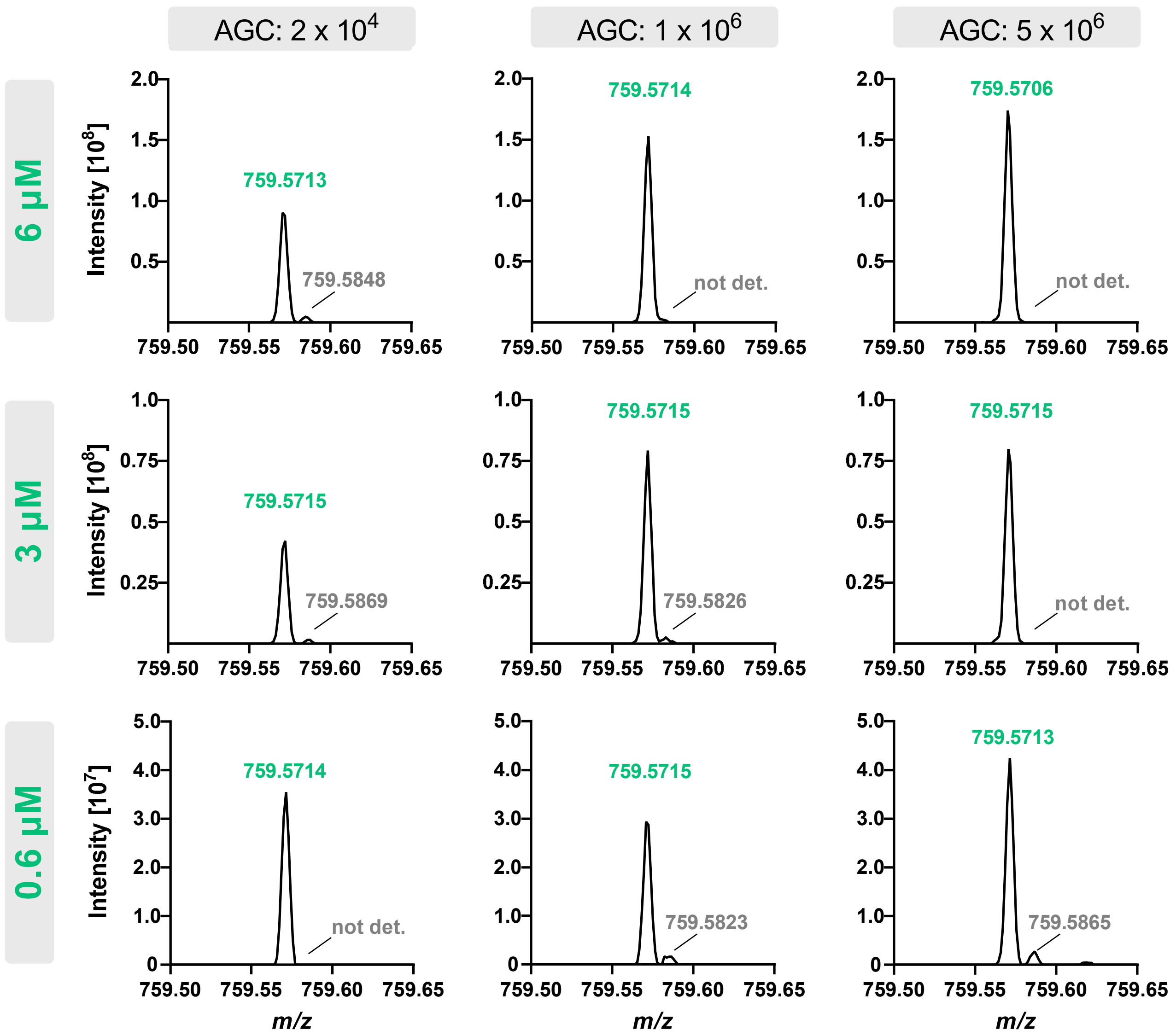

Fig. S6: Concentration and trap-filling dependent interference of PG 15:0/18:1[D7] and PC 16:0/18:2(9Z,12Z) in Orbitrap MS. The signals of [$^{13}C_1$] PC 16:0/18:2(9Z,12Z) (*m/z* 759.5728) are highlighted in green and those of PG 15:0/18:1[D7] (*m/z* 759.5875) in gray. A mixture of the standards was analyzed by FIA-ESI(+)-HRMS at different concentrations keeping a fixed concentration ratio of 12 : 1 (PC : PG). The concentrations of PC 16:0/18:2(9Z,12Z) : PG 15:0/18:1[D7] used are 6 µM : 0.5 µM (**top row**), 3 µM : 0.25 µM (**middle row**), and 0.6 µM : 0.05 µM (**bottom row**). Full MS spectra were recorded using different filling settings of the trap by setting the automatic gain control (AGC) to 2×10^4 (**left column**), 1×10^6 (**middle column**), and 5×10^6 (**right column**).

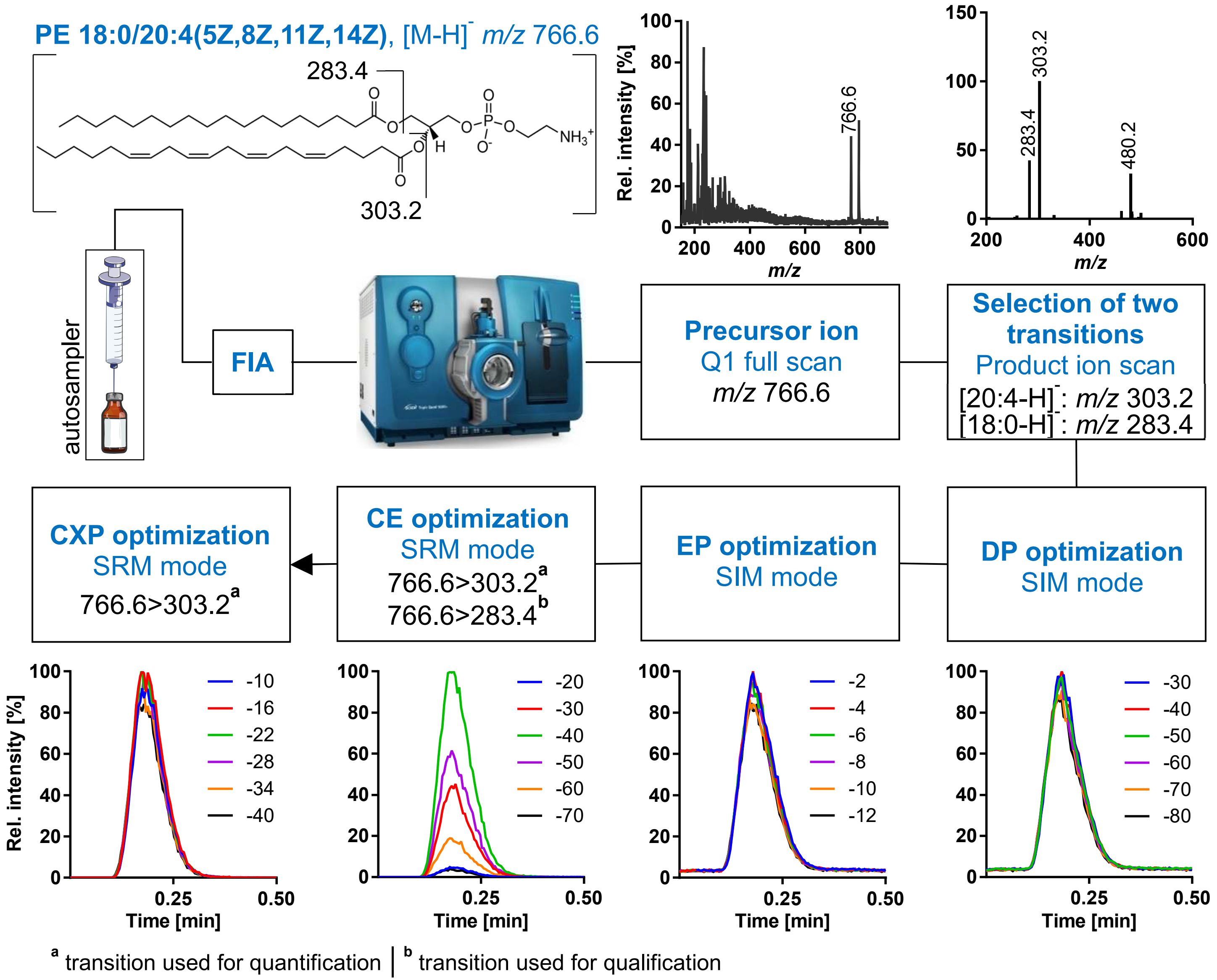

Fig. S7: Method development of the targeted LC-ESI(-)-MS/MS method. Shown is a scheme illustrating the individual steps of optimization including selection of transitions, as well as optimization of electronic parameters, i.e., DP, EP, CE and CXP. Optimization was carried out by flow injection analysis (FIA) from repeated injections of 5 µL of a standard solution. Data is shown exemplary for PE 18:0/20:4(5Z,8Z,11Z,14Z).

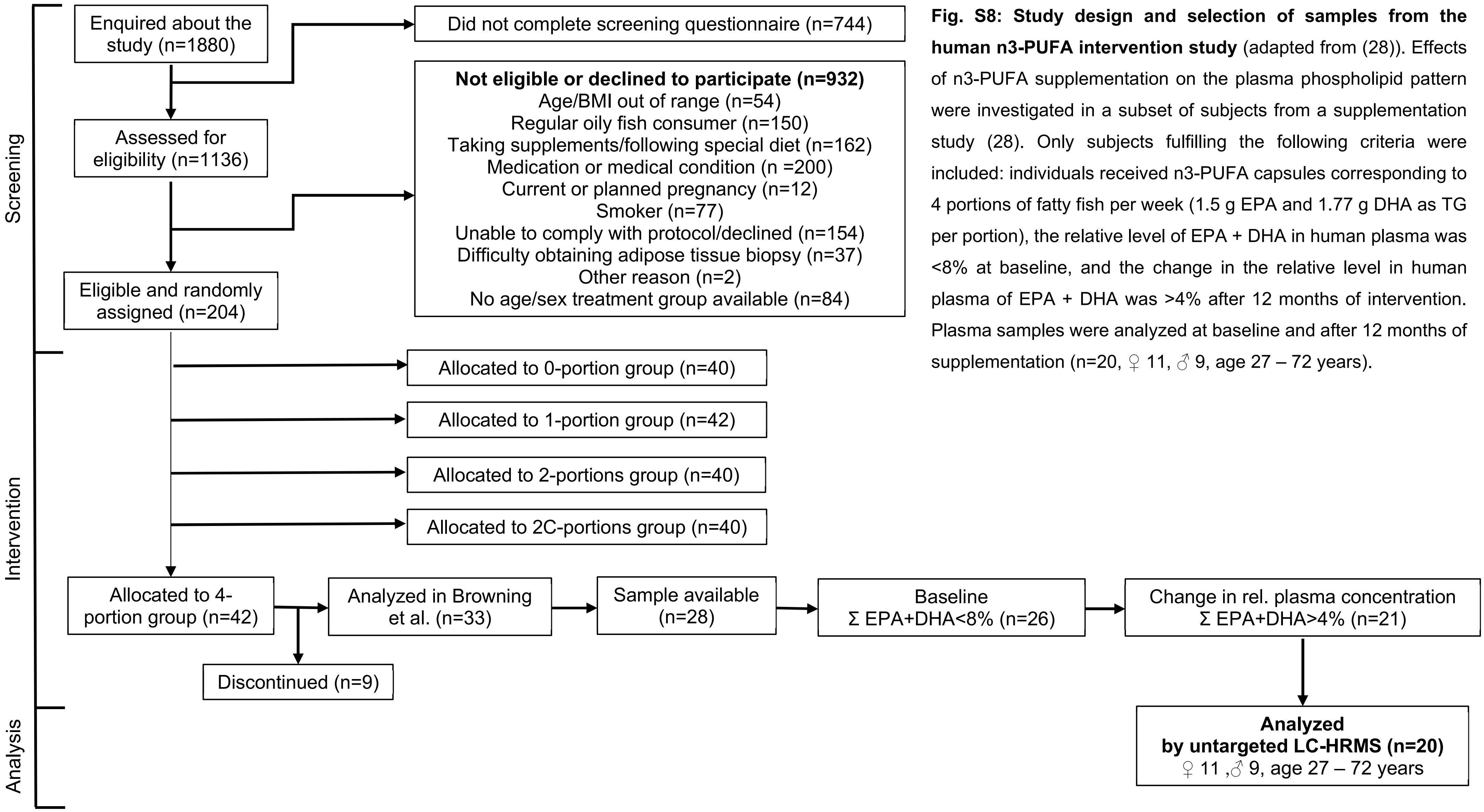

Fig. S8: Study design and selection of samples from the human n3-PUFA intervention study (adapted from (28)). Effects of n3-PUFA supplementation on the plasma phospholipid pattern were investigated in a subset of subjects from a supplementation study (28). Only subjects fulfilling the following criteria were included: individuals received n3-PUFA capsules corresponding to 4 portions of fatty fish per week (1.5 g EPA and 1.77 g DHA as TG per portion), the relative level of EPA + DHA in human plasma was <8% at baseline, and the change in the relative level in human plasma of EPA + DHA was >4% after 12 months of intervention. Plasma samples were analyzed at baseline and after 12 months of supplementation (n=20, ♀ 11, ♂ 9, age 27 – 72 years).

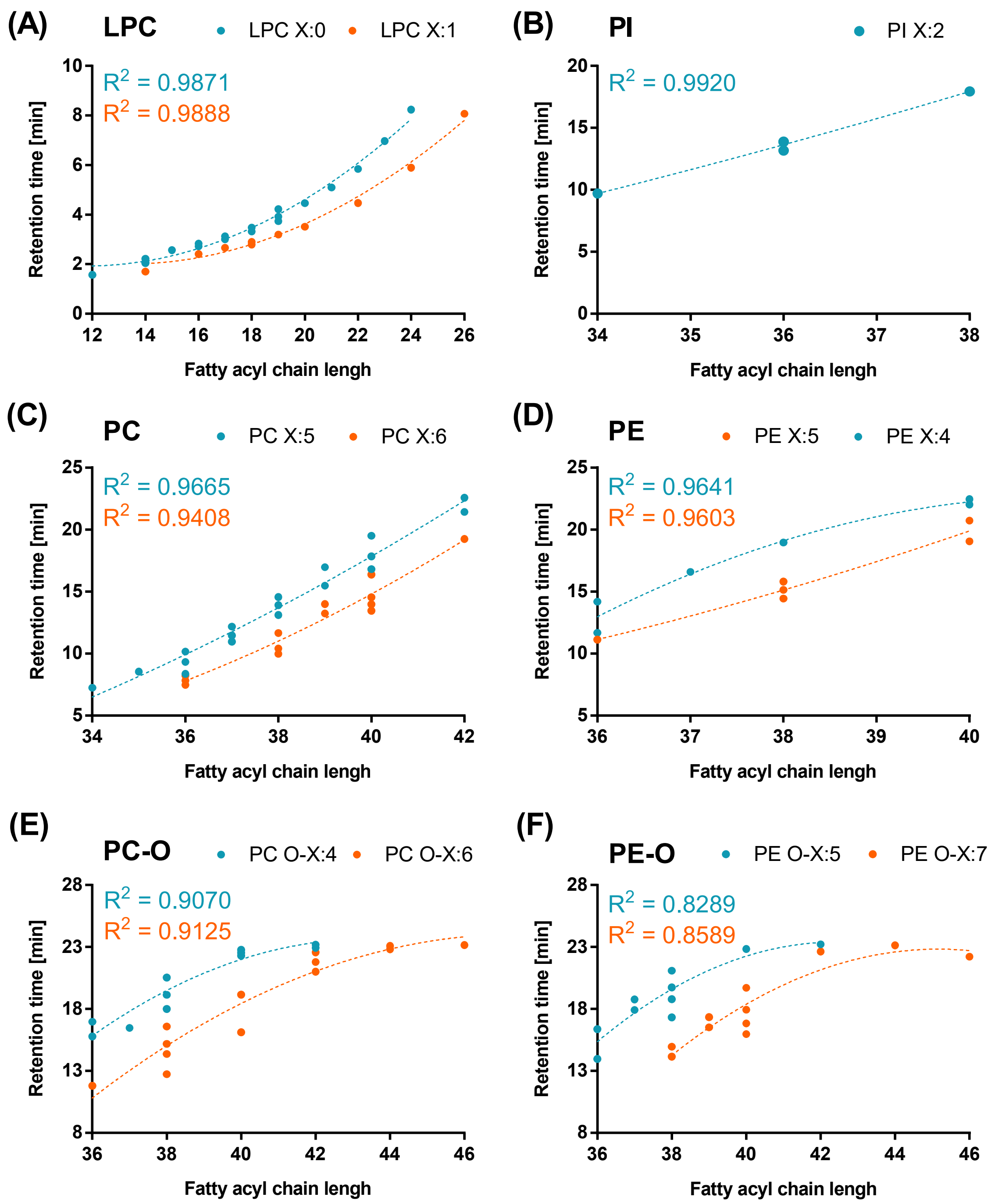

Fig. S9: Polynomial dependences of the retention times on the length of the fatty acyl chains (X = number of carbons of the fatty acyl chains). Shown are **(A)** LPC X:0 and LPC X:1, **(B)** PI X:2, **(C)** PC X:5 and PC X:6, **(D)** PE X:5 and PE X:4, **(E)** PC O-X:4 and PC O-X:6, and **(F)** PE O-X:5 and PE O-X:7.

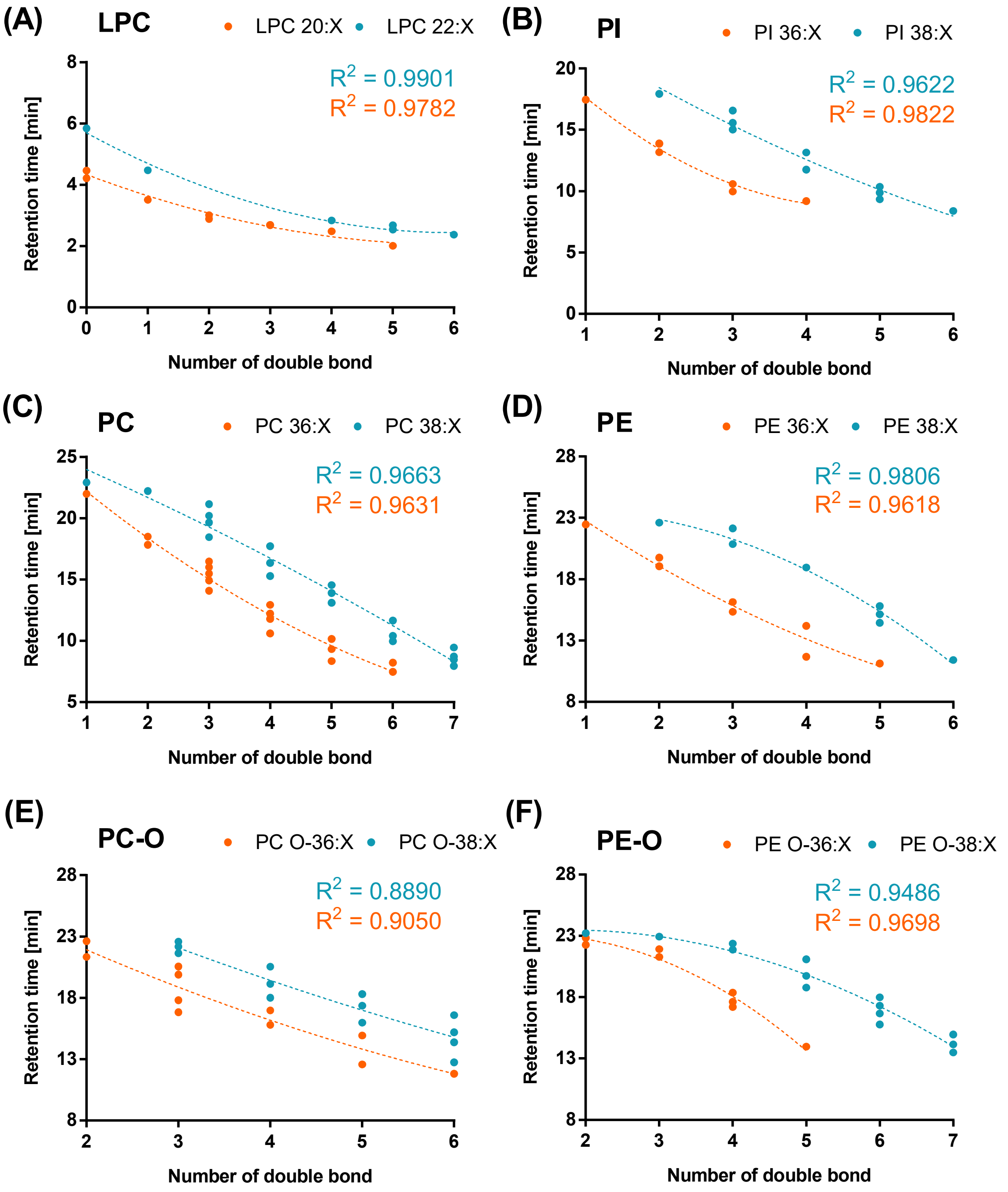

Fig. S10: Polynomial dependences of the retention times on the number of double bonds (X = double bond number). Shown are **(A)** LPC 20:X and LPC 22:X, **(B)** PI 36:X and PI 38:X, **(C)** PC 36:X and PC 38:X, **(D)** PE 36:X and PE 38:X, **(E)** PC O-36:X and PC O 38:X, and **(F)** PE O-36:X and PE O-38:X.

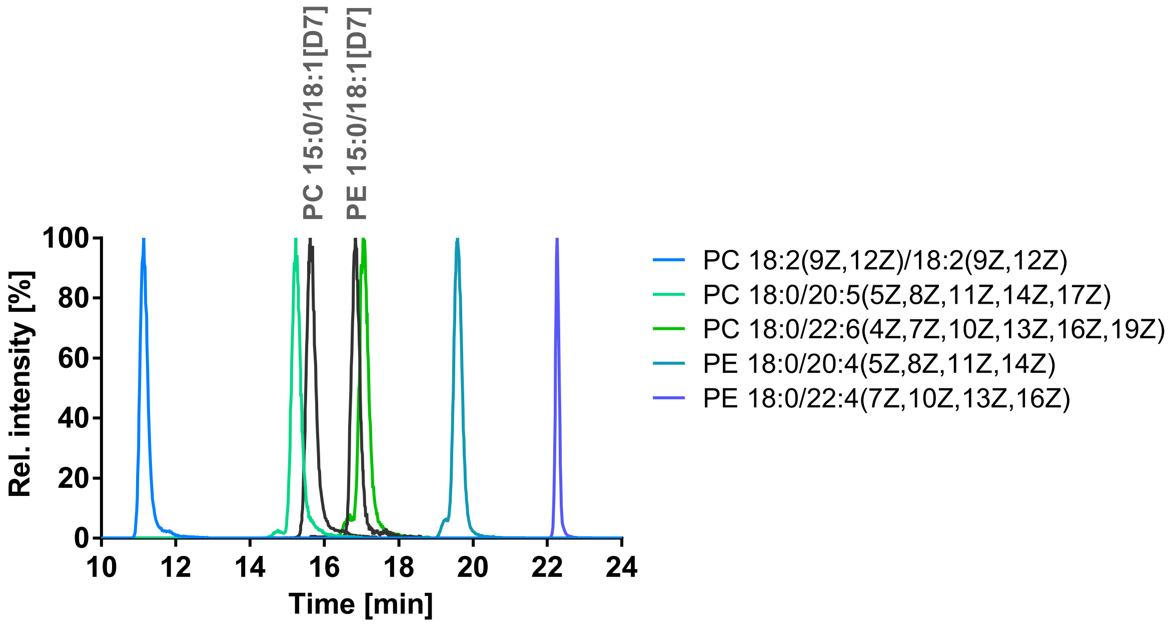

Fig. S11: Chromatographic separation of phospholipids by targeted LC-ESI(-)-MS/MS. Shown are the MRM signals of the quantifier transitions of selected phospholipids as well as the IS for the analysis of a multi-analyte standard solution (400 nM).

References

1. Narváez-Rivas M, Zhang Q. Comprehensive untargeted lipidomic analysis using core–shell C30 particle column and high field orbitrap mass spectrometer. Journal of Chromatography A. 2016;1440:123-34.
2. Hu C, Zhou Y, Feng J, Zhou S, Li C, Zhao S, et al. Untargeted lipidomics reveals specific lipid abnormalities in nonfunctioning human pituitary adenomas. Journal of proteome research. 2019;19(1):455-63.
3. Criscuolo A, Zeller M, Cook K, Angelidou G, Fedorova M. Rational selection of reverse phase columns for high throughput LC–MS lipidomics. Chemistry and physics of lipids. 2019;221:120-7.
4. Khan MJ, Codreanu SG, Goyal S, Wages PA, Gorti SK, Pearson MJ, et al. Evaluating a targeted multiple reaction monitoring approach to global untargeted lipidomic analyses of human plasma. Rapid Communications in Mass Spectrometry. 2020;34(22):e8911.
5. Shan J, Qian W, Kang A, Peng L, Xie T, Lin L, et al. Lipid profile perturbations in the plasma and lungs of mice with LPS-induced acute lung injury revealed by UHPLC-ESI-Q Exactive HF MS analysis. Journal of pharmaceutical and biomedical analysis. 2019;162:242-8.
6. Chen W, Zeng J, Wang W, Yang B, Zhong L, Zhou J. Comprehensive metabolomic and lipidomic analysis reveals metabolic changes after mindfulness training. Mindfulness. 2020;11(6):1390-400.
7. Schwaiger M, Schoeny H, El Abiead Y, Hermann G, Rampler E, Koellensperger G. Merging metabolomics and lipidomics into one analytical run. Analyst. 2019;144(1):220-9.
